# Exploring the Oxidative Stress Mechanism of Buyang Huanwu Decoction in Intervention of Vascular Dementia Based on Systems Biology Strategy

**DOI:** 10.1155/2021/8879060

**Published:** 2021-03-03

**Authors:** Kailin Yang, Liuting Zeng, Anqi Ge, Yaqiao Yi, Shanshan Wang, Jinwen Ge

**Affiliations:** ^1^The First Affiliated Hospital of Hunan University of Chinese Medicine, Changsha, Hunan Province, China; ^2^Key Lab of Hunan Province for Prevention and Treatment of Cardio-Cerebral Diseases with Integrated Traditional Chinese and Western Medicine, Hunan University of Chinese Medicine, Hunan, China; ^3^Department of Rheumatology and Clinical Immunology, Peking Union Medical College Hospital, Chinese Academy of Medical Sciences & Peking Union Medical College, Beijing, China

## Abstract

**Objective:**

To explore the oxidative stress mechanism of modified Buyang Huanwu decoction (MBHD) in intervention of vascular dementia (VD) based on systems biology strategy.

**Methods:**

In this study, through the reverse virtual target prediction technology and transcriptomics integration strategy, the active ingredients and potential targets of MBHD treatment of VD were analyzed, and the drug-disease protein-protein interaction (PPI) network was constructed. Then, bioinformatics analysis methods are used for Gene Ontology (GO) enrichment analysis and pathway enrichment analysis, and finally find the core biological process. After that, in animal models, low-throughput technology is used to detect gene expression and protein expression of key molecular targets in oxidative stress-mediated inflammation and apoptosis signaling pathways to verify the mechanism of MBHD treatment of VD rats. Finally, the potential interaction relationship between MBHD and VD-related molecules is further explored through molecular docking technology.

**Results:**

There are a total of 54 MBHD components, 252 potential targets, and 360 VD genes. The results of GO enrichment analysis and pathway enrichment analysis showed that MBHD may regulate neuronal apoptosis, nitric oxide synthesis and metabolism, platelet activation, NF-*κ*B signaling pathway-mediated inflammation, oxidative stress, angiogenesis, etc. Among them, SIRT1, NF-*κ*B, BAX, BCL-2, CASP3, and APP may be important targets for MBHD to treat VD. Low-throughput technology (qRT-PCR/WB/immunohistochemical technology) detects oxidative stress-mediated inflammation and apoptosis-related signaling pathway molecules. The molecular docking results showed that 64474-51-7, cycloartenol, ferulic acid, formononetin, kaempferol, liquiritigenin, senkyunone, wallichilide, xanthinin, and other molecules can directly interact with NF-*κ*B p65, BAX, BCL-2, and CASP3.

**Conclusion:**

The active compounds of MBHD interact with multiple targets and multiple pathways in a synergistic manner, and have important therapeutic effects on VD mainly by balancing oxidative stress/anti-inflammatory and antiapoptotic, enhancing metabolism, and enhancing the immune system.

## 1. Introduction

Vascular dementia (VD) is a cognitive impairment syndrome caused by a series of cerebrovascular factors, which cause blood circulation disorders and impaired brain function [1]. The results of epidemiology show that by 2040, there will be 81 million dementia patients in developing countries around the world, of which VD patients account for about 30% of the population [2, 3]. The prevalence of VD in Western developed countries is between 1% and 4%, while the prevalence of VD in China has reached 8%, and the incidence of VD among 65-year-olds is rapidly increasing [4, 5]. Finding drugs to prevent and treat VD is one of the important contents explored by medical researchers in the world [6]. Modified Buyang Huanwu decoction (MBHD) consists of *Hedysarum multijugum Maxim*. (Huang Qi), *Angelicae sinensis Radix* [Dang Gui or *Angelica sinensis* (*Oliv*.) *Diels*], *Pheretima Aspergillum* (*E*. *Perrier*) (Di Long or *Pheretima*), *Chuanxiong Rhizoma* (Chuan Xiong or *Ligusticum striatum DC*.), *Acoritataninowii Rhizoma* (Shi Chang Pu or *Acorus calamus* var. *angustatus Besser*), *Panax notoginseng* (*Burk*.) *F*. *H*. *Chen ex C*. *Chow* (San Qi), and *Polygalae Radix* (Yuan Zhi or *Polygala tenuifolia Willd*.) with a ratio of 120 : 15 : 15 : 10 : 10 : 9 : 10 [7]. Clinical studies have shown that MBHD can prevent and interfere with vascular dementia [8], and its mechanism may be achieved by participating in the reconstruction of synapses and enhancing the expression of LTP [8]. The prognosis of VD is related to the underlying disease causing vascular damage and the location of intracranial vascular disease. By improving cerebral circulation and preventing the recurrence of cerebrovascular diseases, symptoms can be reduced and the disease can be prevented from further deterioration [9]. In cerebrovascular diseases, MBHD has a good anti-ischemic effect, which can reduce the area of cerebral infarction in animal models of cerebral ischemia and improve the neurological dysfunction after cerebral ischemia [10]. MBHD can also promote angiogenesis and inhibit neuronal apoptosis and improve the learning and memory abilities of VD rats [11–14]. However, the specific mechanism of MBHD to improve VD needs further study.

Currently, network pharmacology is considered as a new model for drug development [15, 16]. This method can analyze and organize information and data in existing databases (genes, diseases, drug network libraries, etc.), and use professional network analysis software to build a “drug-target-disease” interaction network [17]. In addition, through the use of network analysis methods, network pharmacology can systematically construct a disease network for drug intervention, comprehensively analyze the relationship between drug targets and disease targets, and evaluate the efficacy and side effects of drugs on this basis. Because traditional Chinese medicine (TCM) has the characteristics of multiple components, multiple targets, and multiple channels, it is also researched on a systematic and overall level, so the research ideas of network pharmacology have the same goals as TCM prescriptions [18]. Network pharmacology provides new ideas for the modernization and development of TCM [19, 20]. Therefore, in this study, bilateral common carotid artery ligation (2-VO) was used to replicate the VD rat model, and the microarray analysis technique (including network pharmacology) was used to explore the protective effect of MBHD on the hippocampal neurons of the VD model rat and its molecular mechanism.

## 2. Materials and Methods

### 2.1. Potential Components and Potential Targets Prediction and VD-Related Gene Collection

The compound information of MBHD comes from TCMSP (http://lsp.nwu.edu.cn/tcmsp.php) [21], TCM@Taiwan (http://tcm.cmu.edu.tw/zh-tw/) [22], and TCMID (http://www.megabionet. org/tcmid/) [23]. The TCMSP database was used to provide compounds related to the pharmacokinetic properties including oral bioavailability (OB), drug-likeness properties (DL), and Caco-2 parameters to predict the components of MBHD [21]. The standard was OB ≥ 30%, Caco − 2 > −0.4, and DL ≥ 0.18. In addition, due to the limitations of pharmacokinetic prediction, this study retrieved a large amount of literature and included oral absorbable pharmacologically active compounds for supplementation [24–27].

The predicted compounds are standardized by PubChem (https://pubchem.ncbi.nlm.nih.gov/) to obtain their SMILES formula. Those SMILES formulas of the components were input into the SwissTargetPrediction Database (http://www.swisstargetprediction.ch/) to predict the potential targets of each component [28]. The OMIM database (http://omim.org/) [29] and GeneCards (http://www.genecards.org) [30] were utilized to collect the VD-related disease genes and targets. In GeneCards, the targets with relevant score ≥ 1.0 were selected for sequence research.

### 2.2. Network Construction and Analysis Methods

In molecular biology, the interactive gene/protein search tool STRING (https://string-db.org/) searches and predicts gene/protein interactions based on biological databases and web resources [31]. Protein-protein interaction (PPI) network is an important tool for the system to understand cellular processes. This network can be used to filter and evaluate functional genomic data, and provide a visual platform for annotating the structure and function of proteins [31]. In this study, the online database STRING was used to obtain the PPI data of MBHD potential targets and VD genes, and Cytoscape v3.7.2 was used to construct a visual PPI network diagram. In the PPI network, the closely connected part of the nodes is called cluster. The cluster module analysis of the PPI network can provide information for studying the pathogenesis of diseases and the mechanism of drugs [32]. In this study, the MCODE, a plug-in of Cytoscape, was used to analyze the PPI networks to detect the clusters. Finally, the genes and targets in clusters or in PPI networks were input into DAVID (https://david-d.ncifcrf.gov) for Gene Ontology (GO) enrichment analysis and pathway enrichment analysis.

### 2.3. Experimental Materials

#### 2.3.1. Experimental Animal

One hundred and twenty (120) male specific pathogen-free (SPF) grade SD rats were purchased from Hunan Slack Jingda Experimental Animal Co., Ltd. (experimental animal production license number is SCXK (xiang) 2013-0004), weighing 250-300 g and raised in the Experimental Animal Center of Hunan University of Chinese Medicine. The breeding environment is at room temperature (24 ± 1) °C, relative humidity (50 ± 5)%, and 12 h/12 h alternate day and night. The rats started the experiment after 2 weeks of adaptive feeding. The entire experimental procedure was approved by the ethics committee of Hunan University of Chinese Medicine (HCM-00150023).

#### 2.3.2. Experimental Drugs

Modified Buyang Huanwu decoction (MBHD) consists of *Hedysarum multijugum Maxim*. (Huang Qi; specimen number: 2015076211), *Angelicae sinensis Radix* (Dang Gui or *Angelica sinensis* (*Oliv*.) *Diels*; specimen number: 201504212), *Pheretima Aspergillum* (*E*.*Perrier*) (Di Long or *Pheretima*; specimen number: 2015035123), *Chuanxiong Rhizoma* (Chuan Xiong or *Ligusticum striatum DC*.; specimen number: 2015074430), *Acoritataninowii Rhizoma* (Shi Chang Pu or *Acorus calamus* var. *angustatus Besser*; specimen number: 201407210), *Panax notoginseng* (*Burk*.) *F*. *H*. *Chen ex C*. *Chow* (San Qi; specimen number: 201505792), *Polygalae Radix* (Yuan Zhi or *Polygala tenuifolia Willd*.; specimen number: 201504212) with a ratio of 120 : 15 : 15 : 10 : 10 : 9 : 10. All herbs are certified by Associate Professor Liu Lin of Hunan University of Chinese Medicine to be authentic products specified in the 2015 edition of *Pharmacopoeia of the People's Republic of China*. The equivalent dose of MBHD rats is 17.01 g/kg through the conversion of the human/mouse coefficient. Oxiracetam capsules were purchased from CSPC. The clinical equivalent dose for rats is 0.216 g/kg.

#### 2.3.3. Preparation of Drugs

The herbs of MBHD were mixed, soaked in 5 times the volume of distilled water for 2 hours, and boiled for 0.5 hours on strong fire and 1 hour on slow fire. Then, the filtrate was collected, and the filtrate was extracted again with 3 times the volume of distilled water according to the above steps, and the filtrate was mixed twice and concentrated by a rotary evaporator. The final drug concentration was 5.1 g of crude drug/ml.

#### 2.3.4. Instruments and Reagents

Rabbit anti-rat polyclonal antibodies SIRT1, nuclear factor *κ*B which inhibits the protein subunit (I*κ*B*α*), and NF-*κ*B p65 were purchased from Proteintech (Catalog No.: 10268-1-AP, 10268-1-AP, and 10745-1-AP, respectively). Ordinary agarose was purchased from Biomiga, Inc.; TRIzol reagent was purchased from Sigma, Inc.; mRNA reverse transcription kit and SYBR Premix Ex Taq were purchased from Takara, Inc.; BSA was purchased from Huami, Inc.; PVDF membrane was purchased from Amersham, Inc.; HRP-coupled goat anti-rabbit IgG was purchased from Sigma, Inc.; PV-9000 two-step detection kit was purchased from Beijing Zhongshan Jinqiao Biotechnology Co., Ltd.; reverse transcription cDNA kit and SYBR dye were purchased from Takara, Inc. The primers were designed and synthesized by Shanghai Shenggong Biology Co., Ltd. according to the nucleic acid sequence searched by NCBI.

MT-200 Morris water maze was purchased from Chengdu Taimeng Technology Co., Ltd.; Motic B5 image analysis system was purchased from McAudi Industrial Group; PCR amplification machine (2400 PCR system) was purchased from PerkinElmer, Inc.; Eppendorf desktop low-temperature microcentrifuge was purchased from Eppendorf, Inc.; horizontal electrophoresis was purchased from Bio-Rad, Inc.; Gel Doc1000 gel imaging analysis system was purchased from Bio-Rad, Inc.; Mixer Gentus was purchased from MicroBio, Inc.; J2-21 high-speed centrifuge, J6-HC centrifuge GS-15R, and high-speed desktop centrifuge were purchased from Beckman, Inc.; clean bench was purchased from Suzhou Purification Equipment Co., Ltd.; automatic plate washer was purchased from Beijing Pulang New Technology Co., Ltd.; microscopic imaging system of Mike Audi was purchased from Motic, Inc.; CFX96 real-time PCR system was purchased from Bio-Rad, Inc.; RE-5002 rotary evaporator was purchased from Ride Instruments Co., Ltd.; SMART 3.0 Small Animal Behavior Video Collection and Analysis System was purchased from Panlab, Inc.

### 2.4. Experimental Methods

#### 2.4.1. Animal Modeling, Grouping, and Intervention

Twenty rats were randomly selected as the sham operation group by only freeing bilateral common carotid arteries without ligation. The remaining 100 rats underwent bilateral common carotid artery ligation (2-VO) method to replicate the VD rat model according to Reference [33]. The Morris water maze test was used to eliminate unqualified rats after modeling (i.e., the escape latency was less than 5 s or greater than 120 s). After excluding the rats that died during the modeling and administration process and the water maze experiment was unqualified, the successful modeling rats were divided into model groups, MBHD high-, medium-, and low-dose groups, and positive control group, and no less than 15 rats were included in each group. According to the results of the water maze test, 5 mice were selected for gene chip detection in the model group and the MBHD dose group. The remaining rats are used for pathological morphology and molecular biology testing.

Rats in MBHD high-, medium-, and low-dose groups were intragastrically administered with MBHD 17.0, 34.0, and 51.0 g/kg, respectively. Rats in the positive control group were intragastrically administered with oxiracetam 0.216 g/kg. The remaining 3 groups were given equal volume of distilled water. The drug intervention lasted 30 days.

#### 2.4.2. Learning and Memory Ability Test

The learning and memory ability of rats was tested by Morris water maze. The rats were trained for 5 consecutive days. On the 6th day, the water platform was removed, the rats were put into the water from 4 quadrants, and the Motic B5 image analysis system was used to record the number of times the rats crossed the platform in 120 s.

#### 2.4.3. Histopathological Observation of the Hippocampus

HE staining was used to detect histopathological changes in the hippocampus of rats. The hippocampal tissue samples of each group of rats were fixed with 4% paraformaldehyde and dehydrated by ethanol gradient, sliced, stained, and finally observed under a microscope.

#### 2.4.4. Agilent mRNA Expression Profiling Chip Experiment

The freshly collected rat hippocampus tissue was placed in a 1.5 ml EP tube, wrapped with gauze, quickly frozen in liquid nitrogen at −70°C, placed in a sufficient amount of dry ice, and sent to Beijing Boao Jingdian Biotechnology Co., Ltd. for mRNA extraction, purification, and detection. The qualified RNA was detected by Agilent mRNA chip hybridization, and image scanning and data analysis were performed to obtain the differential expression information of mRNAs between the two groups.

#### 2.4.5. Quantitative Real-Time PCR (qRT-PCR)

The total RNA of the rat hippocampus was extracted by the TRIzol method. Total RNA is reverse transcribed into cDNA according to the instructions of the reverse transcription cDNA kit. The qRT-PCR analysis was performed using the CFX96 real-time PCR system according to the method described in the kit instructions, and the specific primers are shown in Table 1.

#### 2.4.6. SIRT1, I*κ*B*α*, NF-*κ*B p65, Bax, Bcl-2, and Caspase-3 Protein Expression Detected by Immunohistochemistry

After the rat hippocampus tissue was fixed with 4% paraformaldehyde, section deparaffinized, 3% hydrogen peroxide treatment, antigen restoration, etc., the primary antibody (1 : 200) was added and placed overnight at 4°C, and then, HRP-IgG secondary antibody (1 : 400) was added. Finally, after operations such as color development, dehydration, and mounting, 5 fields of view were randomly selected under a light microscope, and the average gray value of the Motic image analysis system was used to determine the average gray value. The smaller the gray value, the higher the positive protein expression.

#### 2.4.7. SIRT1, I*κ*B*α*, NF-*κ*B p65, Bax, Bcl-2, and Caspase-3 Protein Expression Detected by Western Blot

100 mg of tissue was added to 200 *μ*l RIPA lysis buffer and 4 *μ*l PMSF. The tissue was ground and centrifuged under low temperature conditions, and the supernatant was collected. After collecting the protein, the protein concentration was measured with a microplate reader, and the protein concentration was calculated according to the standard curve. Then, SDS-PAGE electrophoresis, film transfer, and other operations were carried out. After ECL color development, observation and photographing are carried out in the gel imaging system. The image analysis software IPP6.0 is used to analyze the integral optical density value of the protein.

### 2.5. MBHD Quality Control via Reversed Phase High-Performance Liquid Chromatography (RP-HPLC)

#### 2.5.1. Sample Preparation

MBHD sample: MBHD 6.94 ml was adjusted to 10.0 ml with water. Standard sample: ferulic acid standard 9.4 mg was placed in a 100 ml volumetric flask and dissolved with methanol-1% glacial acetic acid (1 : 1) and diluted to the mark. Negative control solution: after Chuanxiong Rhizoma in MBHD was removed, other herbs were prepared as a negative control solution according to the MBHD preparation method.

#### 2.5.2. RP-HPLC Condition

Chromatography column is Hypersi1 C18 (200 mm × 4.6 mm, 5 *μ*m). Mobile phase is methanol-1% glacial acetic acid (36 : 64). Column temperature is 25°C. Flow rate is 1 ml/min. Detection wavelength is 322 nm.

#### 2.5.3. Standard Curve Drawing

Standard solution 1 ml was diluted to 10 ml, and then according to the above chromatographic conditions, each injection volume was 2 *μ*l, 4 *μ*l, 8 *μ*l, 12 *μ*l, 16 *μ*l, and 20 *μ*l. The regression equation of the standard curve was obtained with the peak area as *y* and the injection volume as *x*(ng): *y* = 3.782*x* − 4.4(*r* = 0.9995, *n* = 6) and the linear range: 18.8 ng-188 ng. Ferulic acid: tR = 9.40 ± 0.12 min, RSD = 1.4%(*n* = 8), peak area precision RSD = 2.0%(*n* = 4) (Table 2 and Figure S1).

### 2.6. Statistical Analysis

Statistical analysis was performed using SPSS 17.0. Measurement data are expressed as the mean ± standard deviation (*x* ± *s*). The sample is tested for the homogeneity of variance first. When the variance is uniform, the one-way ANOVA test is used for analysis, and the LSD method is used for multiple comparisons between groups. When the variance is not uniform, the nonparametric rank sum test is used for analysis. The Kruskal-Wallis *H* test is used to compare the total difference, and then, the Mann–Whitney *U* test is used to compare the two groups.

### 2.7. Molecular Docking Analysis

The molecular structure of each compound was searched in PubChem (http://scifinder.cas.org), and the structure of the compound was reconstructed in ChemBioDraw strictly according to the structure on PubChem. These compounds are then saved in the “mol2” file format and their energy is minimized. The PDB database (https://www.rcsb.org/) is used to retrieve the 3D structure of SIRT1, NF-*κ*B p65 (RELA), BAX, BCL-2, and CASP3, and download the file in the “pdb” format [34]. Discovery Studio Client ver. 4.5 software is used to hydrogenate proteins, remove water, and remove ligand molecules. AutoDock ver. 4.2 software is used to convert compound molecules and protein molecules into “pdbqt” format, and finally run Vina for molecular docking. If the binding energy is less than 0, the compound (ligand) and protein (receptor) can bind spontaneously. The binding energy ≤ −5.0 kcal/mol is considered that the ligand can bind to the receptor stably.

## 3. Results and Discussion

### 3.1. Research Results of RP-HPLC

#### 3.1.1. Precision Test and Repeatability Test

Precision test: Standard solution (0.094 mg/ml) of 5 *μ*l was taken, repeated injection 5 times; according to the above chromatographic conditions, the peak area was determined (Table 3).

Repeatability test: 5 copies of MBHD were randomly taken out, 5 *μ*l each, and the content of ferulic acid was determined according to the above chromatographic conditions (Table 4 and Figure S1).

#### 3.1.2. Recovery Test

Six copies of MBHD sample were added to standard products 1.6 ml, 2.0 ml, and 2.4 ml, respectively. Then, 5 *μ*l of each was taken, and the content of ferulic acid was determined according to the above chromatographic conditions (Table 5).

#### 3.1.3. Sample Determination

Three samples were processed in parallel, each sample was injected twice, each injection volume was 5 *μ*l, the HPLC chart and peak area were obtained, and the content of ferulic acid (mg/100 g crude drug) was determined according to the external standard method (see Table 6 and Figure S2). It can be calculated that the content of ferulic acid is 1.40 ± 0.03 mg/g.

### 3.2. Potential Components and Targets and VD Genes

**Figure 1:**
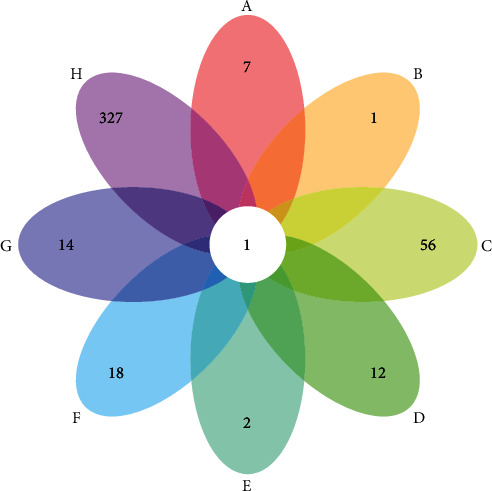
Venn diagram of MBHD potential targets and VD genes (A: Chuanxiong Rhizoma; B: Angelicae sinensis Radix; C: Hedysarum multijugum Maxim.; D: Earthworm Lumbricus; E: Panax notoginseng (Burk.) F. H. Chen ex C. Chow; F: Polygala tenuifolia; G: Acoritataninowii Rhizoma; H: VD genes).

There are a total of 54 MBHD components, 252 potential targets, and 360 VD genes. The MBHD potential components are 1,7-dihydroxy-3,9-dimethoxy pterocarpene, 3,6′-disinapoyl sucrose, 3,9-di-O-methylnissolin, 4-guanidino-1-butanol, 64474-51-7, 64997-52-0, 73340-41-7, 7-O-methylisomucronulatol, 8-isopentenyl-kaempferol, alpha-asarone, astragaloside IV, beta-asarone, beta-sitosterol, bifendate, butylidenephthalide, calycosin, calycosin 7-O-glucoside, cholesteryl ferulate, cycloartenol, diop, eudesmin, ferulic acid, formononetin, guanosine, hederagenin, hyrcanoside, isoflavanone, isorhamnetin, jaranol, kaempferol, ligustilide, liquiritigenin, mairin, mandenol, myricanone, ononin, perlolyrine, polygalasaponin XXVIII, polygalaxanthone III, quercetin, senegin III, senkyunolide I, senkyunone, sibiricaxanthone A, sitosterol, stigmasterol, tenuifolin, tenuifoliose A, tenuifoliose H, tenuifoliose I, tenuifoliside A, tenuifoliside B, wallichilide, and xanthinin. Meanwhile, there are some overlaps between MBHD potential target set and VD gene set (Figure 1). The details of MBHD potential targets and VD genes are shown in Table S1 and S2.

**Figure 2:**
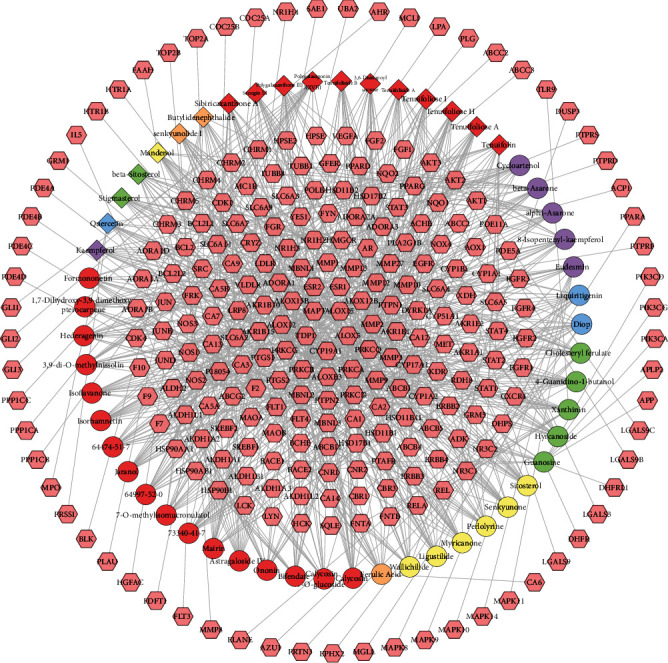
Compound-compound target network of MBHD (pink hexagons stand for compound targets; red, orange, yellow, green, blue, and purple circles stand for compounds of *Hedysarum multijugum Maxim*., *Angelicae sinensis Radix*, *Chuanxiong Rhizoma*, *Earthworm Lumbricus*, *Panax notoginseng* (*Burk*.) *F*. *H*. *Chen ex C*. *Chow*, and *Acoritataninowii Rhizoma*, resp.; red diamond stands for compounds of *Polygala tenuifolia*; orange diamond stands for common compounds of *Chuanxiong Rhizoma* and *Angelicae sinensis Radix*; yellow diamond stands for common compounds of *Chuanxiong Rhizoma* and *Panax notoginseng* (*Burk*.) *F*. *H*. *Chen ex C*. *Chow*; green diamond stands for common compounds of *Angelicae sinensis Radix* and *Panax notoginseng* (*Burk*.) *F*. *H*. *Chen ex C*. *Chow*; blue diamond stands for common compounds of *Hedysarum multijugum Maxim*. and *Panax notoginseng* (*Burk*.) *F*. *H*. *Chen ex C*. *Chow*; purple diamond stands for common compounds of *Acoritataninowii Rhizoma* and *Panax notoginseng* (*Burk*.) *F*. *H*. *Chen ex C*. *Chow*).

The relationship of MBHD potential targets and potential components was shown in compound-compound target network of MBHD. This network contains 306 nodes (252 compound target nodes and 54 compound nodes) and 808 edges. In this network, nodes closing to the center show more interactions with compounds than peripheral nodes. This indicates that many targets are regulated by multiple compounds, but some can be modulated by only one compound (peripheral nodes, such as HPSE, HPSE2, HSP90AA1, HSP90AB1, and HSP90B1) (Figure 2).

### 3.3. MBHD-VD PPI Network Analysis

#### 3.3.1. MBHD-VD PPI Network

**Figure 3:**
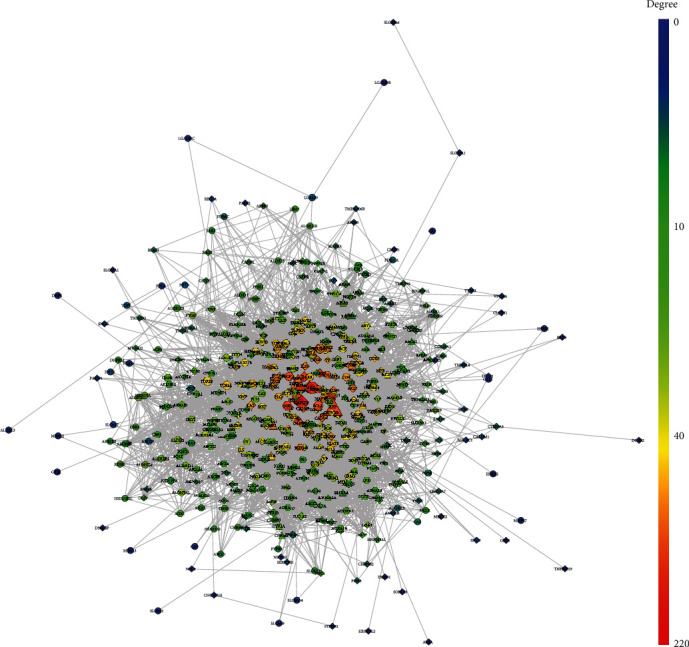
MBHD-VD PPI network (circles stand for MBHD targets; diamonds stand for VD genes; triangles stand for MBHD-VD targets, respectively. The color of the nodes was related to the degree. The size of the nodes was positively related to their betweenness).

The MBHD potential targets, VD genes, and their PPI data were input into Cytoscape to construct MBHD-VD PPI network. This network is composed of 512 nodes (213 compound targets, 267 VP genes, and 32 BHD-VP targets) and 8468 edges (Figure 3). The targets are arranged in descending order of degree. The top 20 targets can be divided into 3 categories: (1) MBHD targets: AKT1 (190 edges), SRC (176 edges), JUN (161 edges), PIK3CA (157 edges), EGFR (150 edges), BCL2 (148 edges), PIK3CG (136 edges), PIK3CD (131 edges), and HSP90AA1 (127 edges); (2) VD genes: GAPDH (220 edges), MAPK3 (169 edges), IL6 (168 edges), MAPK1 (164 edges), TNF (163 edges), IGF1 (134 edges), CALM2 (134 edges), CALM1 (129 edges), and CASP3 (125 edges); and (3) MBHD-VD targets: APP (166 edges) and VEGFA (163 edges).

#### 3.3.2. Biological Processes of MBHD-VD PPI Network

**Figure 4:**
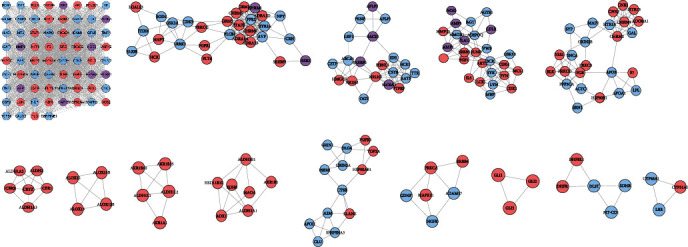
Clusters of MBHD-VD PPI network (pink, blue, and purple circles stand for MBHD targets, VD targets, and MBHD-VD target, respectively).

The MBHD-VD PPI network was analyzed by MCODE, and fourteen clusters were obtained (Table 7 and Figure 4). The targets in the clusters were input into DAVID to perform GO enrichment analysis and get a lot of biological processes.

**Figure 5:**
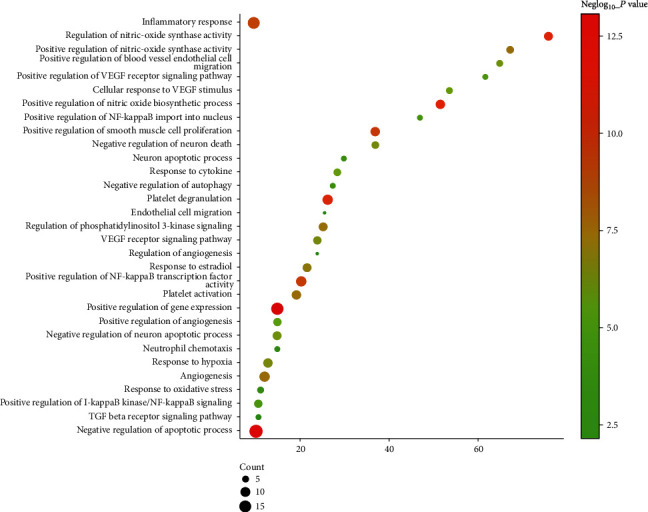
Bubble chart of biological processes in cluster 1 (*x*-axis stands for fold enrichment).

Cluster 1 is related to neuronal apoptosis, nitric oxide synthesis and metabolism, platelet activation, NF-*κ*B signaling pathway-mediated inflammation, oxidative stress, and angiogenesis. Cluster 2 is related to vasoconstriction, cell proliferation, and synaptic transmission. Cluster 3 is related to the differentiation of foam cells and the anabolism of cholesterol. Cluster 4 is related to cell proliferation, platelet activation, and vasodilation. Cluster 5 is related to synaptic transmission, triglyceride, and other lipid metabolisms. Clusters 6, 8, 9, and 14 are related to redox. Cluster 10 is related to apoptosis, platelet degranulation, neuronal apoptosis, synaptic transmission, and coagulation. Cluster 11 is related to apoptosis and nerve cell migration. Clusters 7, 12, and 13 failed to return any VD-related biological processes. The biological processes of cluster 1 are shown in Figure 5 as an example. The details of them are shown in Table S3.

#### 3.3.3. Signaling Pathway of MBHD-VD PPI Network

**Figure 6:**
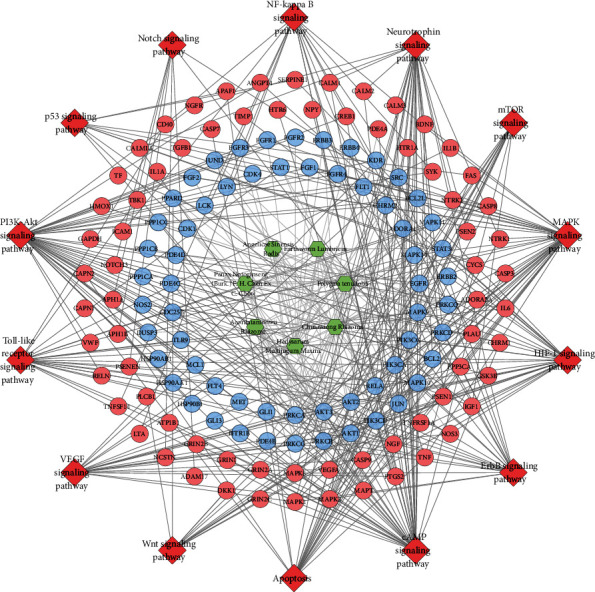
Signaling pathway of MBHD-VD PPI network (blue and pink circles stand for compound target and VP genes, resp; red diamond stands for pathway; green hexagon stands for herb).

**Figure 7:**
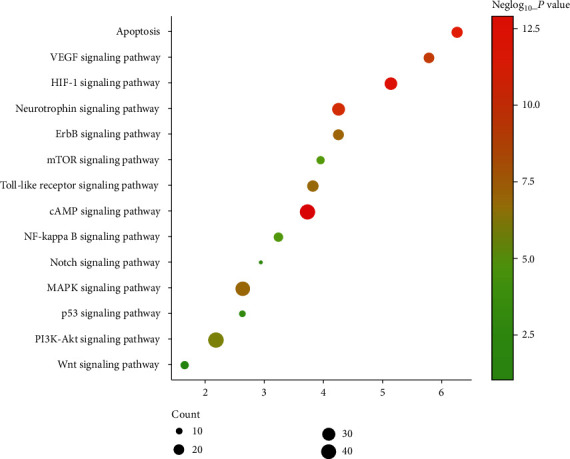
Bubble chart of signaling pathway (*x*-axis stands for fold enrichment).

The targets and genes in MBHD-VD PPI network were input into DAVID to perform pathway enrichment analysis and return fourteen core VP-related pathways (Figure 6). The top 10 signaling pathways are the cAMP signaling pathway, HIF-1 signaling pathway, apoptosis, neurotrophin signaling pathway, VEGF signaling pathway, ErbB signaling pathway, MAPK signaling pathway, Toll-like receptor signaling pathway, PI3K-Akt signaling pathway, and mTOR signaling pathway (Figure 7). The details of the signaling pathway are shown in Table S4.

In this network, *Hedysarum multijugum Maxim*. totally regulate 29 targets (which is the most), while *Chuanxiong Rhizoma* regulate 28 targets. *Earthworm Lumbricus* regulate 16 targets; *Panax notoginseng* (*Burk*.) *F*. *H*. *Chen ex C* regulate 10 targets; *Polygala tenuifolia* control 18 targets; *Acoritataninowii Rhizoma* control 10 targets; *Angelicae sinensis Radix* regulate 12 targets. This once again suggests that *Hedysarum multijugum Maxim*. plays a major role in BHD intervening VP.

TCM network pharmacology is based on computer network modeling analysis and target prediction. It analyzes the mechanism of TCM at the system level and reflects its overall thinking [35]. In recent years, many scholars have used network pharmacology methods to study the treatment of VD with traditional Chinese medicine. Luo et al. established a traditional Chinese medicine-compound-target-signal pathway interaction network model to analyze the mechanism of action of BHD in the treatment of neurodegenerative diseases, and reported that BHD mainly treats neurodegenerative diseases through anti-inflammatory and antiapoptotic pathways [36]. Fang et al. excavated the top 10 VD-resistant plants reported by PubMed from the literature, and explained the anti-VD mechanism of these Chinese medicines from multiple angles based on systematic pharmacological analysis [37]. These studies provide a lot of valuable information reference for exploring the mechanism of TCM to prevent and treat VD. This study used network pharmacological analysis methods to predict the active ingredients and targets of MBHD, constructed the MBHD-VD PPI network, and explored the mechanism of MBHD treatment of VD. In this research, a total of 54 MBHD components, 252 potential targets, and 360 VD genes were found. In the results, one compound can act on multiple targets, and different compounds can act on the same target, which fully confirms the multitarget effect of MBHD. By constructing a compound-target network, it was found that mairin, 64474-51-7, jaranol, isorhamnetin, hederagenin, formononetin, calycosin, bifendate, astragaloside IV, 73340-41-7, 7-O-methylisomucronulatol, 64997-52-0, and 3,9-di-O-methylnissolin can regulate more targets, indicating that these compounds may be important compounds for MBHD to exert pharmacological effects. Among them, jaranol has strong antioxidant capacity [38]. Isorhamnetin, as one of the most important active ingredients of flavonoids, has a wide range of pharmacological activities, such as protecting cardiovascular and cerebrovascular, antitumor, anti-inflammatory, antioxidant, organ protection, and obesity prevention [39, 40]. Hederagenin, as a naturally occurring triterpene, induces (drug-resistant head and neck cancer (HNC) cell apoptosis) resistant HNC cell death through the Nrf2-ARE antioxidant pathway [41]. In animal models of cerebral ischemia/reperfusion injury, hederagenin exerts antiapoptotic and anti-inflammatory effects through the MAPK and NF*κ*B pathways [42]. Formononetin has antioxidant and neuroprotective effects [43]. It may inhibit proinflammatory NF-*κ*B signaling and enhance anti-inflammatory Nrf-2/HO-1 signaling, thereby improving cognitive function [44]. Calycosin has anti-inflammatory, antitumor, and neuroprotective effects. Studies have found that its specific anti-inflammatory mechanism in the treatment of neurodegenerative diseases is related to the TLR/NF-*κ*B and MAPK pathways [45]. In an in vitro model of cerebral ischemia and reperfusion, calycosin can prevent oxidative stress and neuronal apoptosis of HT22 cells through the SIRT1/FOXO1/PGC-1*α* pathway, thereby reducing OGD/R-induced damage [46]. As the main active substance of *Hedysarum multijugum Maxim*., astragaloside IV has a variety of pharmacological effects, including anti-inflammatory, antifibrosis, antioxidative stress, antiasthma, antidiabetic, and immune regulation [47]. The signaling pathways involved include the EGFR-Nrf2 signaling pathway, NF-*κ*B signaling pathway, signaling pathway Nrf2 antioxidant signaling pathway, PI3K/Akt/mTOR signaling pathway, and many other signaling pathways [48].

Meanwhile, the further research (GO and pathway enrichment analysis) showed that the mechanism of MBHD to treat VD is mainly related to neuronal apoptosis, nitric oxide synthesis and metabolism, platelet activation, NF-*κ*B signaling pathway-mediated inflammation, oxidative stress, angiogenesis, etc. Current studies have shown that the pathogenesis of VD mainly includes (1) hypoperfusion and hypoxia caused by microvascular circulatory disorders, (2) increased blood-brain barrier permeability caused by endothelial dysfunction, (3) inflammation and oxidative stress damage the nutritional interaction between neurovascular unit cells, (4) ROS and inflammation inhibit the survival of neurons (neuron apoptosis), and (5) the destruction and demyelination of myelin tablets caused by the oxidative and proinflammatory environment caused by cerebral ischemia and hypoperfusion and the breakdown of the blood-brain barrier [1, 49, 50].

### 3.4. General Condition

The normal group and sham operation group: Before the operation, the rats had bright hair, red lips, bright red eyes, vigorous spirit, frequent fighting, and strong appetite. Within 3 days after the operation, the spirits were sluggish, less active, and appetite decreased, but gradually recovered, the mental state became better, the movement was agile, the voluntary activities increased, and the hair, lips, nails, eyeballs, appetite, etc. recovered to a good state.

The model group: Before modeling, the experimental rats' hair, lips, nails, eyeballs, spirit, and appetite were generally in good condition. After the model was created, various degrees of lethargy, slow response, slow movement, dull fur, dark purple lip nails, increased sleep, and poor appetite appeared. Some rats have different degrees of drooping eyelids, diminished eye fissures, and slight sunken eyeballs; a small number of rats are irritable, more active, irritable, have unstable walking, and turning in circles. Moreover, the self-cleaning ability of rats is reduced, and the hair becomes sparse, dry, and erected or lacks gloss, and occasionally, there are transient paroxysmal convulsions in rats. In some cases, the abdomen gradually swelled into a spherical shape, accompanied by obvious reduction in eating and defecation, rough and dull coat color, gradual weight loss of limbs, and death.

The treatment groups: In the early stage after the successful modeling of the rat, the general condition and behavior of the rat are similar to the VD model group. After a period of drug administration, compared with the model group, the diet, spirit, and activity gradually recovered, and the general situation was better than that of the model group.

### 3.5. Effect of MBHD on the Learning and Memory Ability of VD Rats

**Figure 8:**
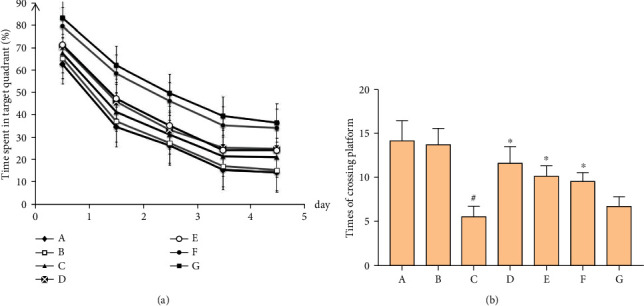
Effect of MBHD on the learning and memory ability of VD rats. (a) Positioning navigation experiment. (b) Space exploration experiment. A: normal group; B: sham operation group; C: model group; D: positive group; E: MBHD high-dose group; F: MBHD medium-dose group; G: MBHD low-dose group. Compared with sham operation group, ^#^*P* < 0.05; compared with model group, ^∗^*P* < 0.05.

1. Positioning navigation experiment: the escape latency of rats in each group was shortened with the extension of training time. There was no significant difference between the blank group and the sham operation group in the escape latency at the same time point (*P* > 0.05). There was a significant difference in the escape latency between the model group and the sham operation group at the same time point (*P* < 0.05), indicating that the model was successful. Compared with the model group, the escape latency in the positive group and the MBHD high- and medium-dose groups at the same time point has significant differences (*P* < 0.05), suggesting that MBHD and oxiracetam can improve learning and memory in VD rats
2. Space exploration experiment: the number of crossing platforms in the model group was significantly reduced (*P* < 0.05). Compared with the model group, the number of times the rats crossed the platform was significantly increased (*P* < 0.05) in the oxiracetam group and the MBHD high- and medium-dose groups, suggesting that the memory ability of the rats was improved after drug intervention (Figure 8)

### 3.6. Effect of MBHD on the Pathological Morphology of the Hippocampus in VD Rats

**Figure 9:**
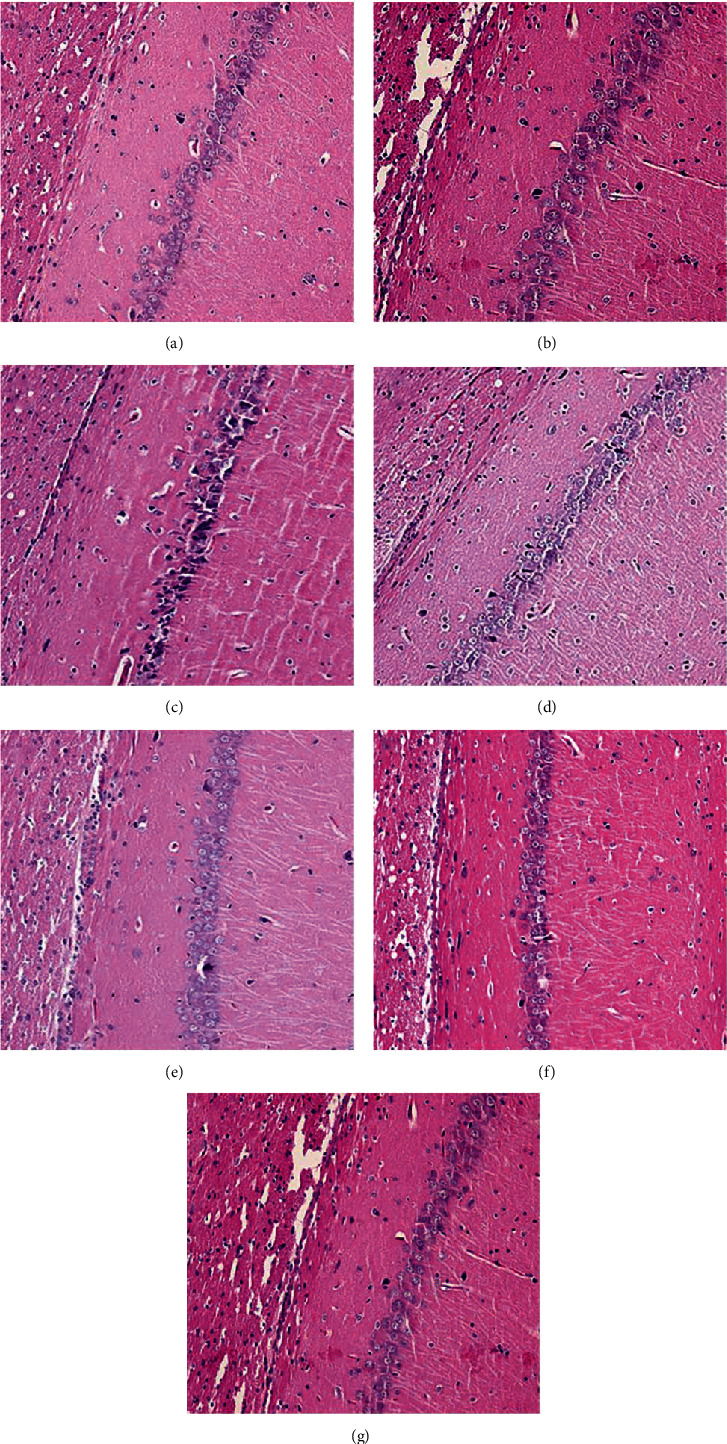
Pathological changes of the hippocampus in VD rats (HE staining, ×200). (a) Normal group. (b) Sham operation group. (c) Model group. (d) Positive group. (e) MBHD high-dose group. (f) MBHD medium-dose group. (g) MBHD low-dose group.

The results of HE staining showed that hippocampal neurons in the model group were degeneration and necrosis (as indicated by the arrow), and inflammatory cell infiltration increased. Compared with the model group, the hippocampal neuron pyramidal cells of the oxiracetam group and the MBHD high-, medium-, and low-dose groups were arranged more neatly, the outline was clear, and the inflammatory cell infiltration was significantly reduced (Figure 9).

### 3.7. Differentially Expressed mRNA Analysis

#### 3.7.1. Differentially Expressed mRNA Results

**Figure 10:**
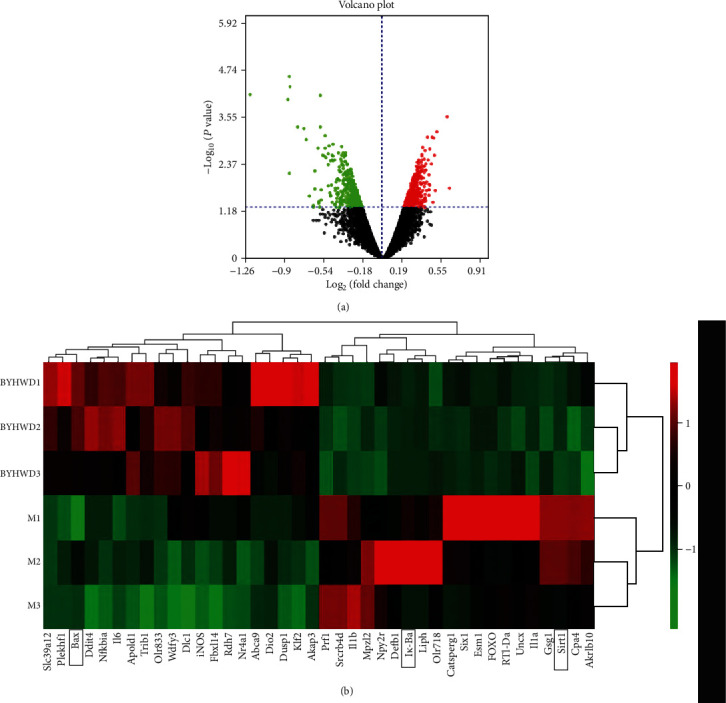
Agilent mRNA profiling chip detection results. (a) Volcanic map of differentially expressed mRNAs. (b) Cluster heat map of differentially expressed mRNAs.

Agilent mRNA was used to detect the mRNA expression profile of rats in the MBHD group and model group. The sample similarity is reflected by sample principal component analysis (PCA). The PCA results show that the overall variance contribution rate is 51.17%.The data of the drug intervention group is relatively clustered (except GZ18), and the data of the model group is relatively divergent. Through the analysis of the two sets of gene chip data, a total of 469 differentially expressed mRNAs were screened, of which 180 were upregulated and 289 were downregulated. Among them, the most upregulated was Slc39a12 (FC = 3.874), and the most downregulated was Liph (FC = 5.986). The differentially expressed mRNA volcano map is shown in Figure 10(a), and the top 20 differentially expressed mRNA cluster heat maps are shown in Figure 10(b).

#### 3.7.2. Protein-Protein Interaction Analysis

**Figure 11:**
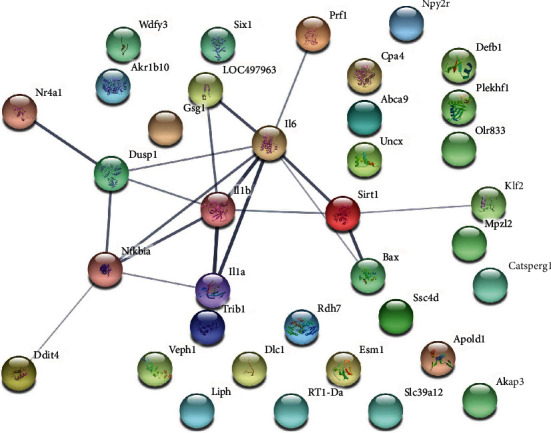
PPI network of differentially expressed mRNA.

The top 20 upregulated and downregulated mRNAs were input into STRING to obtain their PPI data and construct a PPI network (Figure 11), and they were input into DAVID for GO and pathway enrichment analysis. The results showed those mRNAs are related to acute inflammatory response, apoptotic process, ectopic germ cell programmed cell death, response to lipopolysaccharide, positive regulation of apoptotic process, cellular response to organic cyclic compound, extrinsic apoptotic signaling pathway in the absence of ligand, graft-versus-host disease, prion diseases, inflammatory bowel disease (IBD), leishmaniasis, influenza A, type I diabetes mellitus, etc. (Table S5).

### 3.8. Effect of MBHD on the Expression of VD-Related Proteins and mRNA by Immunohistochemistry

#### 3.8.1. Effect of MBHD on the Expression of SIRT1, I*κ*B*α*, and NF-*κ*B p65 Protein in the Hippocampus

**Figure 12:**
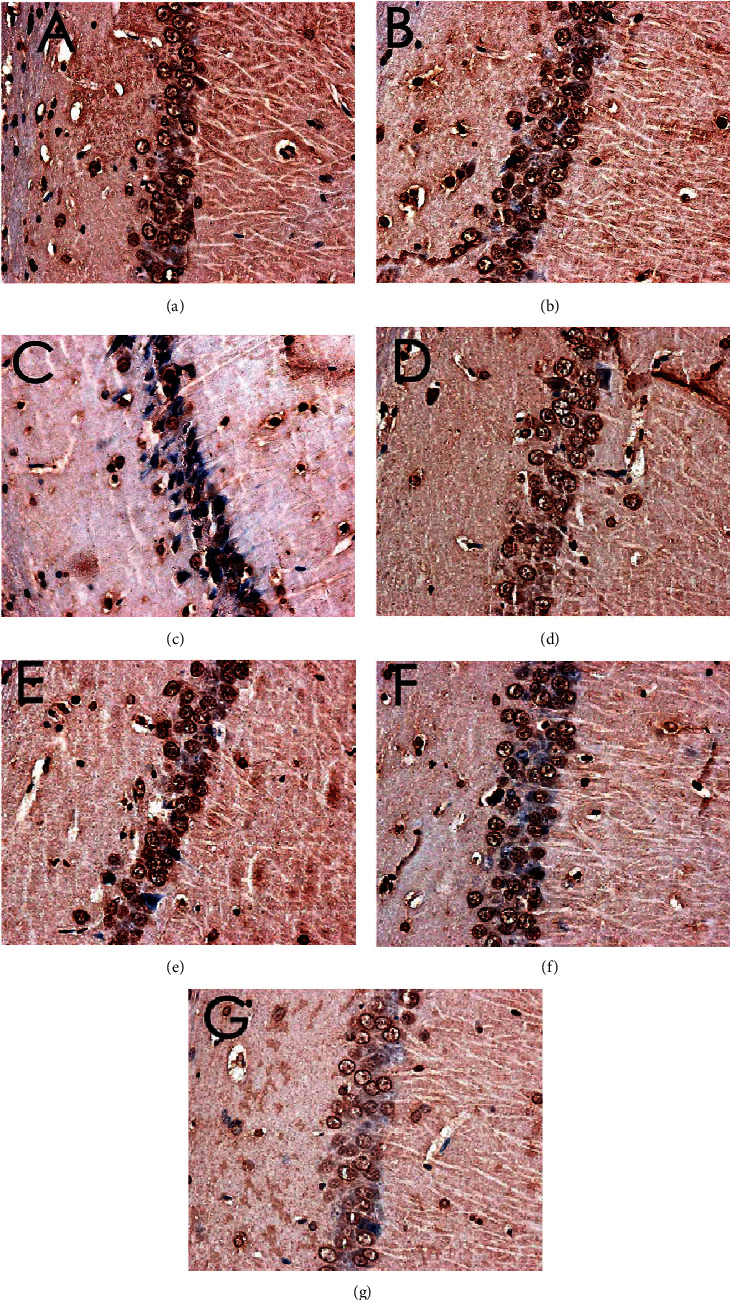
Expression of SIRT1 (immunohistochemistry, ×400). (a) Normal group. (b) Sham operation group. (c) Model group. (d) Positive group. (e) MBHD high-dose group. (f) MBHD medium-dose group. (g) MBHD low-dose group.

**Figure 13:**
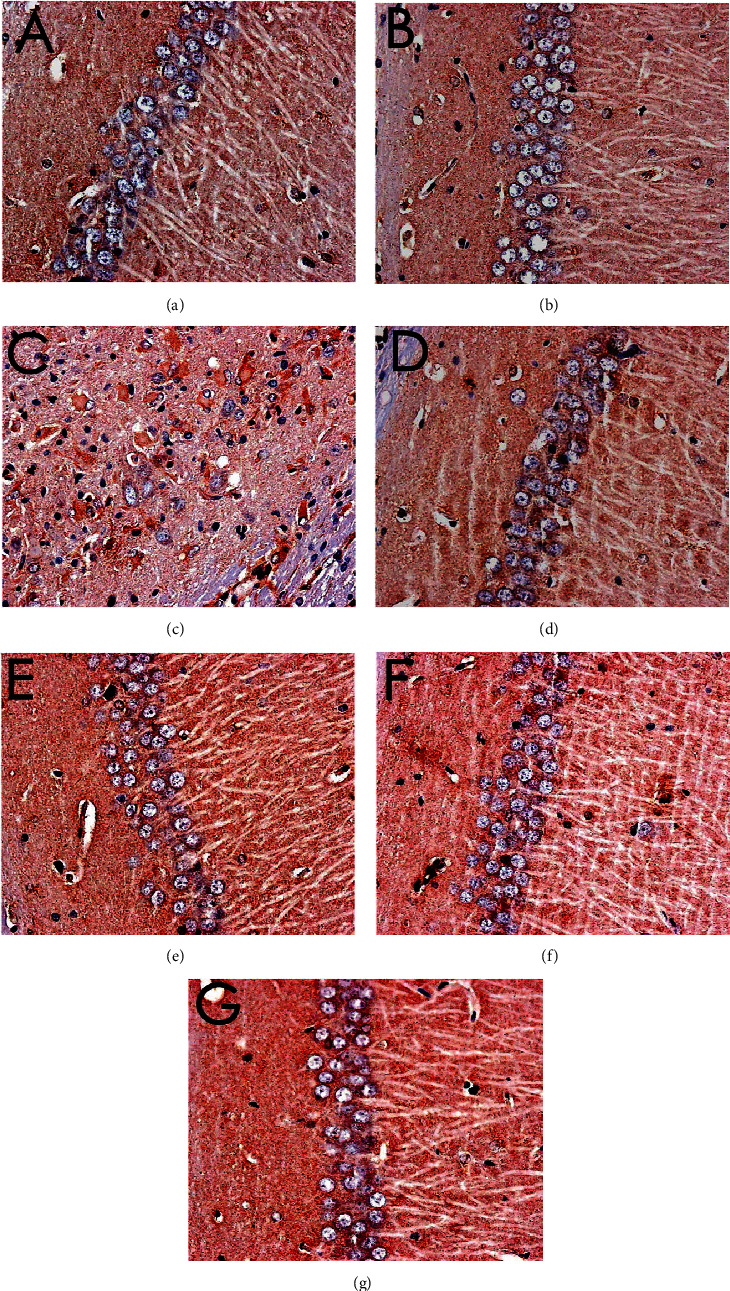
Expression of NF-*κ*B p65 (immunohistochemistry, ×400). (a) Normal group. (b) Sham operation group. (c) Model group. (d) Positive group. (e) MBHD high-dose group. (f) MBHD medium-dose group. (g) MBHD low-dose group.

**Figure 14:**
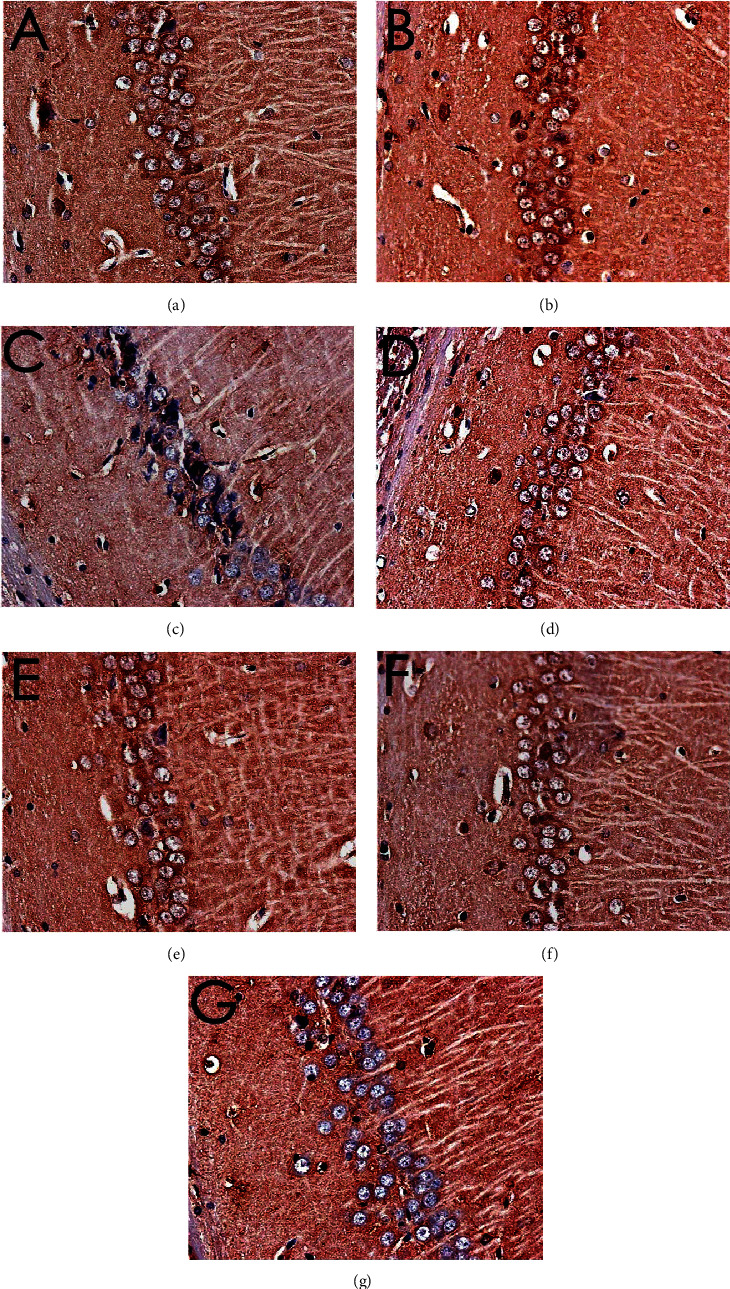
Expression of I*κ*B*α* (immunohistochemistry, ×400). (a) Normal group. (b) Sham operation group. (c) Model group. (d) Positive group. (e) MBHD high-dose group. (f) MBHD medium-dose group. (g) MBHD low-dose group.

**Figure 15:**
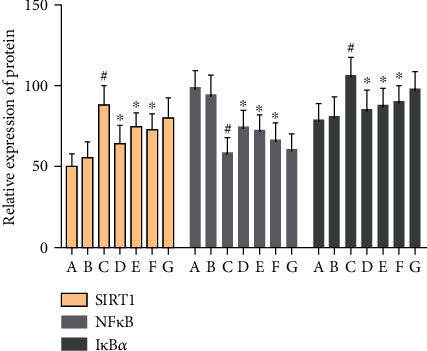
Effect of MBHD on the expression of SIRT1, I*κ*B*α*, and NF-*κ*B p65 protein (A: normal group; B: sham operation group; C: model group; D: positive group; E: MBHD high-dose group; F: MBHD medium-dose group; G: MBHD low-dose group. Compared with the sham operation group, ^#^*P* < 0.05; compared with model group, ^∗^*P* < 0.05).

The immunohistochemical results showed that the positive expression of SIRT1, NF-*κ*B p65, and I*κ*B*α* protein was brown or tan. The results showed that the positive expression of SIRT1 and I*κ*B*α* in the hippocampus of the model group was decreased, and the positive expression of NF-*κ*B p56 was significantly increased (*P* < 0.05). After drug intervention, the positive expression of SIRT1 and I*κ*B*α* increased, and the positive expression of NF-*κ*B p56 decreased significantly (*P* < 0.05) (Figures 12–15).

#### 3.8.2. Effect of MBHD on the Expression of Bax, Bcl-2, and Caspase-3 in the Hippocampus

The positive expression of Bax, Bcl2, and caspase-3 protein was brown or tan particles, the positive expression of Bax protein staining was located in the cytoplasm, and Bcl2 and caspase-3 were located in the nuclear membrane or cytoplasm.

**Figure 16:**
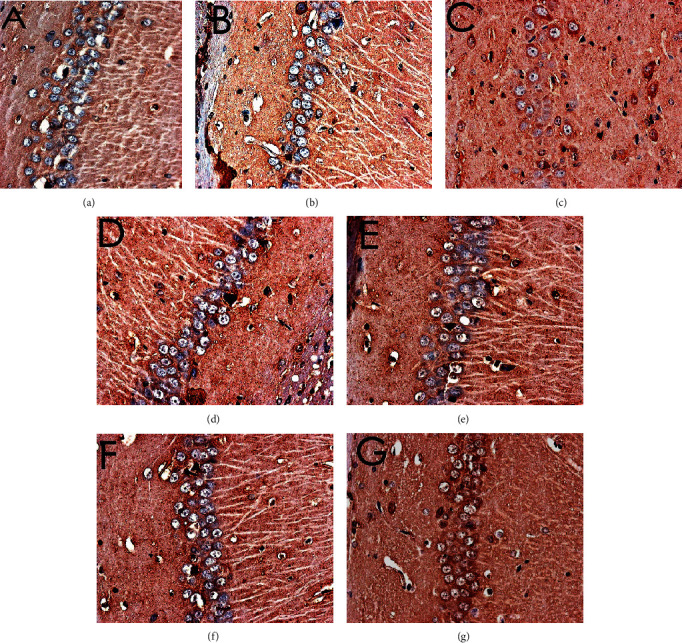
Expression of Bax (immunohistochemistry, ×400). (a) Normal group. (b) Sham operation group. (c) Model group. (d) Positive group. (e) MBHD high-dose group. (f) MBHD medium-dose group. (g) MBHD low-dose group.

**Figure 17:**
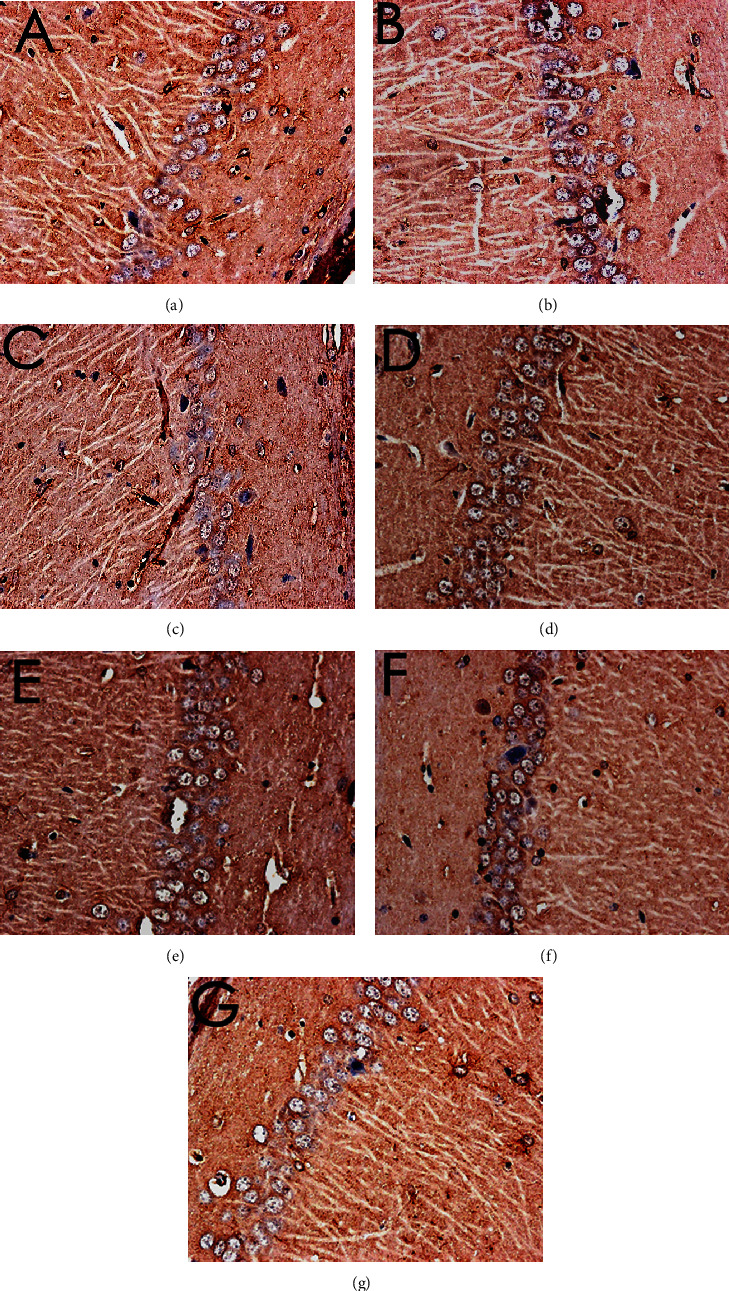
Expression of Bcl-2 (immunohistochemistry, ×400). (a) Normal group. (b) Sham operation group. (c) Model group. (d) Positive group. (e) MBHD high-dose group. (f) MBHD medium-dose group. (g) MBHD low-dose group.

**Figure 18:**
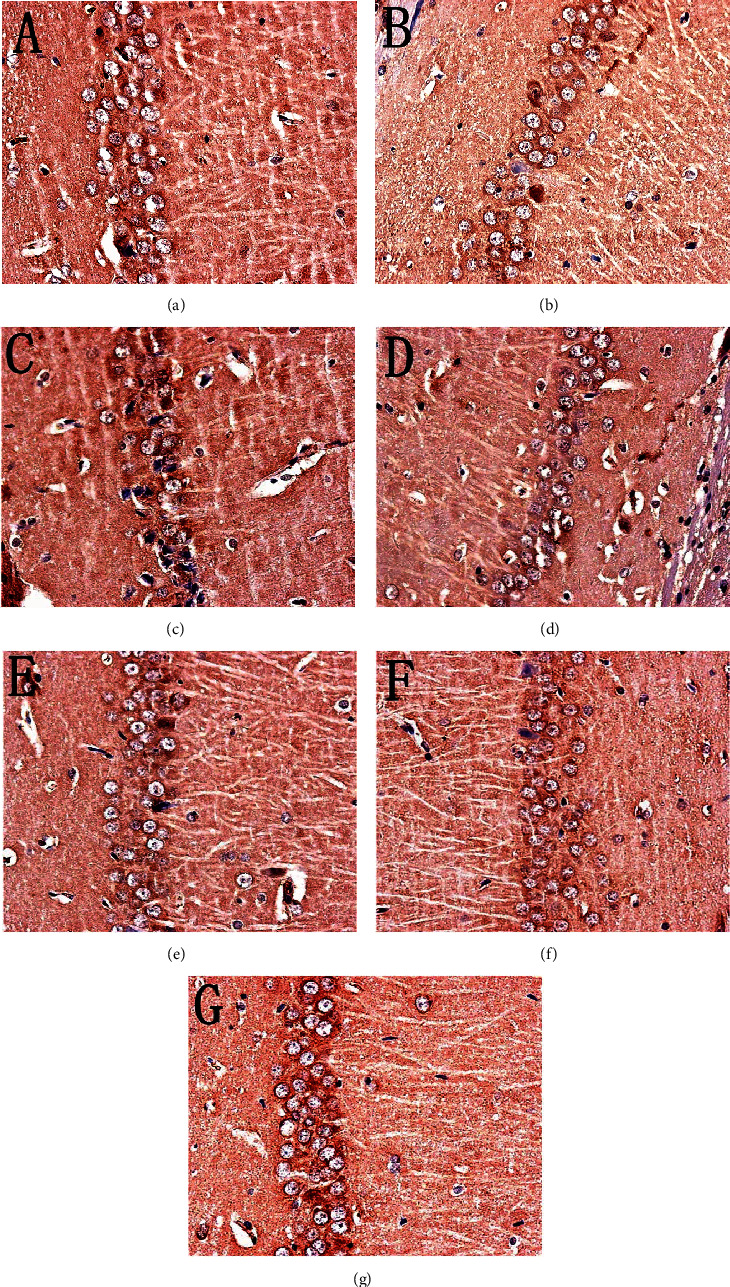
Expression of caspase-3 (immunohistochemistry, ×400). (a) Normal group. (b) Sham operation group. (c) Model group. (d) Positive group. (e) MBHD high-dose group. (f) MBHD medium-dose group. (g) MBHD low-dose group.

**Figure 19:**
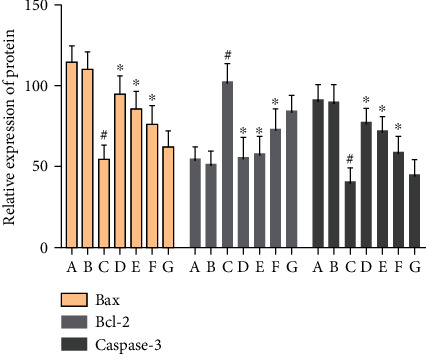
Effect of MBHD on the expression of Bax, Bcl-2, and caspase-3 protein (A: normal group; B: sham operation group; C: model group; D: positive group; E: MBHD high-dose group; F: MBHD medium-dose group; G: MBHD low-dose group. Compared with sham operation group, ^#^*P* < 0.05; compared with model group, ^∗^*P* < 0.05).

There was no significant difference in the expression of Bax-, Bcl-2-, and caspase-3-positive cells in the hippocampus of the normal group compared with the sham operation group (*P* > 0.05). The Bcl-2-positive cells expressed in the hippocampus of VD rats were significantly decreased (*P* < 0.05), while the Bax- and caspase-3-positive cells were significantly increased (*P* < 0.05). After drug intervention, Bcl-2-positive cells increased significantly (*P* < 0.05); Bax- and caspase-3-positive cells decreased significantly (*P* < 0.05) (Figures 16–19).

#### 3.8.3. Effect of MBHD on Expression of SIRT1, NF-*κ*B, and I*κ*B*α* mRNA in the Hippocampus

**Figure 20:**
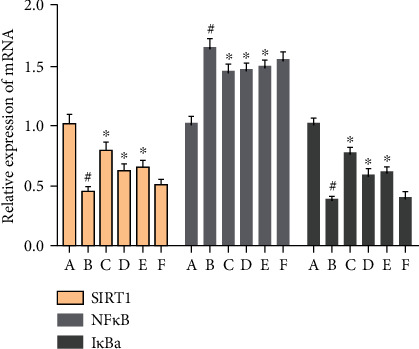
Effect of MBHD on expression of SIRT1, NF-*κ*B, and I*κ*B*α* mRNA (A: sham operation group; B: model group; C: positive group; D: MBHD high-dose group; E: MBHD medium-dose group; F: MBHD low-dose group. Compared with sham operation group, ^#^*P* < 0.05; compared with model group, ^∗^*P* < 0.05).

Compared with the sham operation group, the level of SIRT1 and NF-*κ*B mRNA in the hippocampus of the model group was significantly decreased (*P* < 0.05), while the level of I*κ*B*α* mRNA was significantly increased (*P* < 0.05). After drug intervention, the levels of SIRT1 and I*κ*B*α* mRNA in the hippocampus increased significantly (*P* < 0.05), and the level of NF-*κ*B mRNA decreased significantly (*P* < 0.05) (Figure 20).

#### 3.8.4. Effect of MBHD on Expression of Bax, Bcl-2, and Caspase-3 mRNA in the Hippocampus

**Figure 21:**
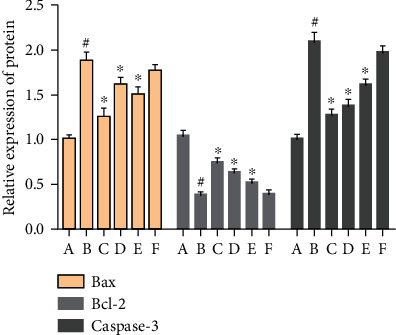
Effect of MBHD on the expression of Bax, Bcl-2, and caspase-3 mRNA (A: sham operation group; B: model group; C: positive group; D: MBHD high-dose group; E: MBHD medium-dose group; F: MBHD low-dose group. Compared with sham operation group, ^#^*P* < 0.05; compared with model group, ^∗^*P* < 0.05).

The level of Bcl-2 mRNA in the hippocampus of the model group was significantly reduced, while the levels of Bax and caspase-3 mRNA were significantly increased (*P* < 0.05). After drug intervention, the levels of SIRT1 and Bcl-2 mRNA expressed in the hippocampus were significantly increased, and the levels of Bax and caspase-3 mRNA were downregulated (*P* < 0.05) (Figure 21).

### 3.9. Effect of MBHD on the Expression of VD-Related Proteins by Western Blot

#### 3.9.1. Effect of MBHD on the Expression of SIRT1, I*κ*B*α*, and NF-*κ*B p65 Protein in the Hippocampus

**Figure 22:**
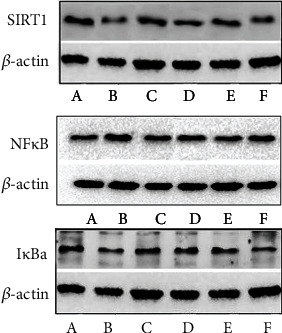
Effect of MBHD on the expression of SIRT1, I*κ*B*α*, and NF-*κ*B p65 protein (A: sham operation group; B: model group; C: positive group; D: MBHD high-dose group; E: MBHD medium-dose group; F: MBHD low-dose group).

**Figure 23:**
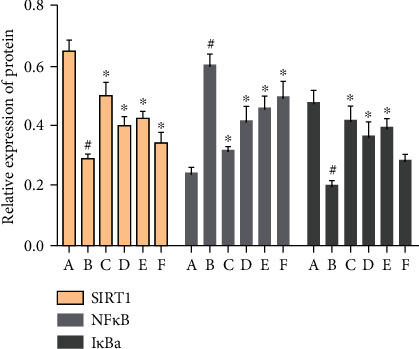
Effect of MBHD on the expression of SIRT1, I*κ*B*α*, and NF-*κ*B p65 protein (A: sham operation group; B: model group; C: positive group; D: MBHD high-dose group; E: MBHD medium-dose group; F: MBHD low-dose group. Compared with sham operation group, ^#^*P* < 0.05; compared with model group, ^∗^*P* < 0.05).

Compared with the sham operation group, there were significant differences in the protein levels of SIRT1, NF-*κ*B, and I*κ*B*α* in the model group (*P* < 0.05), further suggesting the success of the model. After drug intervention, SIRT1 and I*κ*B*α* protein levels increased significantly (*P* < 0.05), and NF-*κ*B protein levels decreased significantly (*P* < 0.05) (Figures 22 and 23).

#### 3.9.2. Effect of MBHD on the Expression of Bax, Bcl-2, and Caspase-3 in the Hippocampus

**Figure 24:**
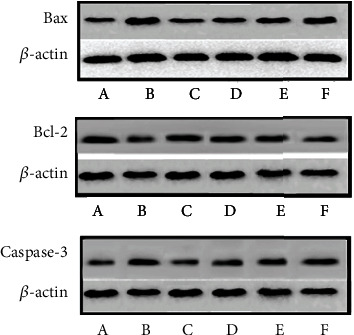
Effect of MBHD on the expression of Bax, Bcl-2, and caspase-3 protein (A: sham operation group; B: model group; C: positive group; D: MBHD high-dose group; E: MBHD medium-dose group; F: MBHD low-dose group).

**Figure 25:**
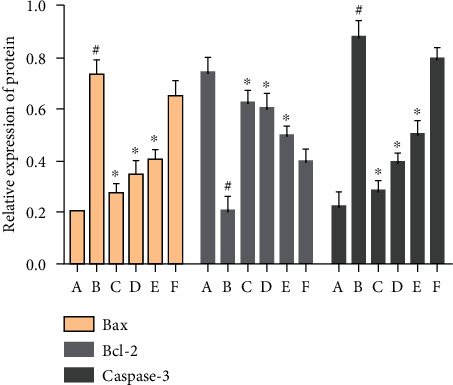
Effect of MBHD on the expression of Bax, Bcl-2, and caspase-3 protein (A: sham operation group; B: model group; C: positive group; D: MBHD high-dose group; E: MBHD medium-dose group; F: MBHD low-dose group. Compared with sham operation group, ^#^*P* < 0.05; compared with model group, ^∗^*P* < 0.05).

Compared with the sham operation group, the Bcl-2 protein level in the hippocampus of the model group was significantly reduced, while the Bax and caspase-3 protein levels were significantly increased (*P* < 0.05). After drug intervention, the level of Bcl-2 protein expressed in the hippocampus increased significantly, and the protein levels of Bax and caspase-3 decreased significantly (*P* < 0.05) (Figures 24 and 25).

### 3.10. Molecular Docking Results of MBHD Compound and SIRT1, RELA, BAX, BCL2, and CASP3

Due to the limitations of the prediction database, this study used molecular docking technology to further explore whether the compounds of MBHD can directly interact with SIRT1, NF-*κ*B p65 (RELA), BAX, BCL-2, and CASP3 molecules. It is generally believed that when the conformation of ligand and receptor is stable, the lower the energy, the greater the possibility of interaction between ligand and receptor. The compounds in the compound-compound target network of MBHD are arranged in descending order of degree, and the top compound of each herb is randomly selected. Finally, 15 compounds (64474-51-7, astragaloside IV, cycloartenol, ferulic acid, formononetin, hyrcanoside, kaempferol, ligustilide, liquiritigenin, mairin, mandenol, quercetin, senkyunone, wallichilide, and xanthinin) were randomly selected for molecular docking with SIRT1, RELA, BAX, BCL2, and CASP3 (Table 8).

**Figure 26:**
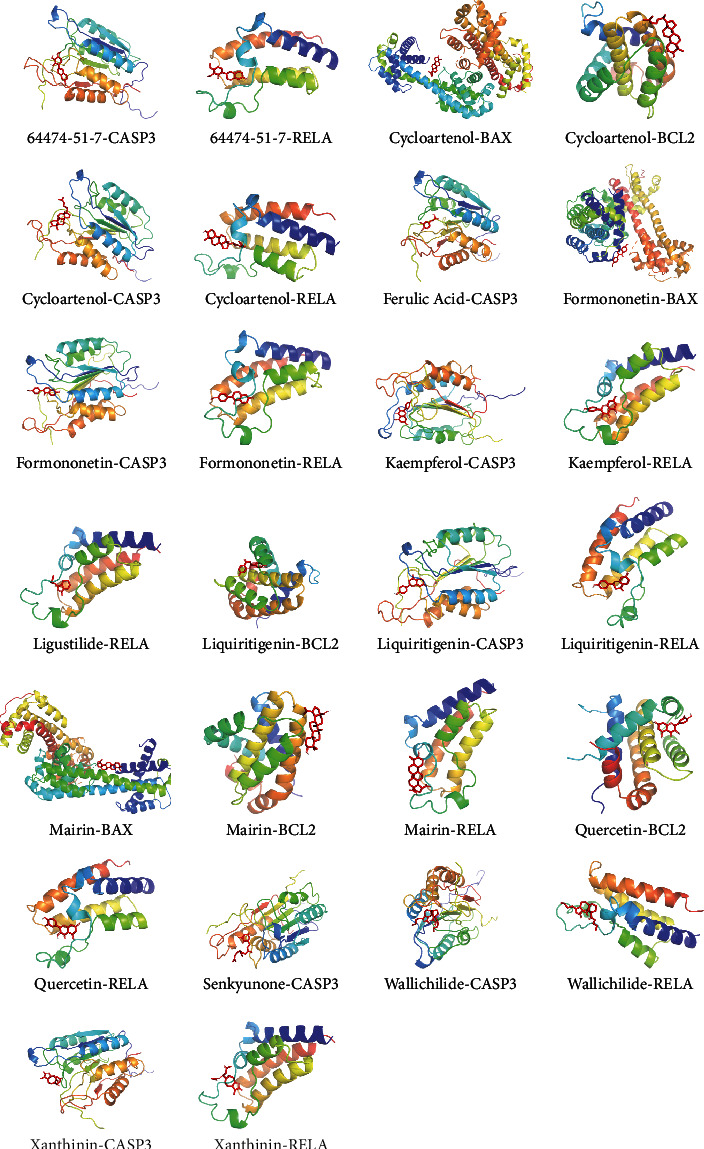
Stable ligand-receptor molecule.

In Table 8, the binding energy of the ligand and the receptor is less than -5 kcal is considered to interact. BAX may interact directly with cycloartenol, formononetin, and mairin. BCL2 may interact directly with cycloartenol, liquiritigenin, mairin, and quercetin. CASP3 may interact directly with 64474-51-7, cycloartenol, ferulic acid, formononetin, kaempferol, liquiritigenin, senkyunone, wallichilide, and xanthinin. NF-*κ*B p65 may interact directly with 64474-51-7, cycloartenol, formononetin, kaempferol, ligustilide, liquiritigenin, mairin, quercetin, wallichilide, and xanthinin (Figure 26). SIRT1 may not directly interact with the 15 small molecules mentioned above, and it may be indirectly regulated by MBHD compounds by interacting with other proteins.

VD refers to a syndrome of learning, memory, and cognitive dysfunction caused by various cerebrovascular diseases. Its clinical characteristics are easy to forget when encountering things, emotional disturbance, and misunderstanding [51]. The pathogenesis of VD is currently unknown. Studies have found that it is closely related to cerebral ischemia and hypoxia and subsequent damage to specific nervous tissues [52]. Therefore, chronic cerebral ischemia and hypoxia may be related to the onset of VD. Previous studies have shown that the 2-VO method can induce ischemic and hypoxic damage to the hippocampus, cortex, and other brain tissues, and then induce VD [53], and the method is easy to operate and reproducible. It has been widely used in VD research [53]. Meanwhile, based on our previous research foundation, we also use this mature model for research on MBHD intervention in VD.

Current reports show that learning and memory and cognitive impairment are the main clinical manifestations of VD. Decreased learning and memory ability is the early clinical manifestation of VD. Effectively improving learning and memory function has become the focus and breakthrough point of VD research. SIRT1-mediated signaling pathways are related to the improvement of learning and memory and cognitive functions [54, 55]. SIRT1 mRNA is abundantly expressed in hippocampal neurons, and it is the material basis for neurodegenerative diseases such as learning and memory, nerve regeneration, and Alzheimer's disease [56]. The “longevity gene” SIRT1 has become a potential therapeutic target for AD, and more and more evidence confirms that it has a definite role in improving AD cognitive impairment by inhibiting *β*-amyloid deposition and inflammatory response [57, 58]. Studies have reported that SIRT1 has a protective effect on cognitive function under many physiological and pathological conditions. After knocking out SIRT1 in mice, its synaptic plasticity is impaired, and learning, memory, and cognitive abilities are reduced [59]. Julien et al. found that the mRNA of SIRT 1 in the brain of AD patients was reduced by 29%, and the protein was reduced by 45%, and the level of SIRT 1 was significantly correlated with the overall predeath cognitive score [60]. Resveratrol is a SIRT1 agonist. Dietary addition of resveratrol can improve the cognitive function of elderly mice by increasing the density of hippocampal microvessels and reducing the number of abnormal microvascular endothelial cells in the hippocampus and cortex [61]. Zhang et al. reported that crocin-1 can increase the expression of SIRT1 protein in the hippocampus of hypoxic rats to improve their learning and memory function [62]. The above results suggest that SIRT1 is closely related to cognitive impairment. The results of this experiment showed that the expression of SIRT1 protein was significantly reduced in the model group and the sham operation group; and after drug intervention, the expression of SIRT1 protein was significantly increased. This suggests that MBHD can participate in the prevention and treatment of VD by upregulating the expression of SIRT1.

Recent studies have found that the SIRT1/NF-*κ*B p65 inflammatory pathway and related molecules are involved in the cognitive function of VD rats. Among them, the inflammatory response plays an important role in the secondary nerve damage after cerebral ischemia, and it participates in the occurrence and development of VD, which has attracted more and more attention. It has been reported that chronic inflammation around plaques and neurofibrillary tangles leads to neurodegenerative diseases [63]. Pathological studies have shown that inflammatory markers are increased in patients with vascular cognitive impairment [63]. NF-*κ*B mediates the inflammatory process of the pathological mechanism of VD. It is the earliest inflammatory factor after cerebral ischemia and a downstream factor of SIRT1. SIRT1 plays an important regulatory role in the inflammatory response by deacetylating inflammatory mediators. NF-*κ*B heterodimeric protein is an important factor regulating the transcription of inflammatory cytokines [64]. I*κ*B*α* is an inhibitor of NF-*κ*B. In the cytoplasm, the combination of the two is in an inactive state. When I*κ*B*α* is stimulated by proinflammatory factors (such as lipopolysaccharide, tumor necrosis factor, and interleukin 1), the inflammatory factors bind to cytokine receptors, and I*κ*B*α* is phosphorylated and then degraded by the ubiquitin-dependent proteasome to promote the transfer of NF-*κ*B into the nucleus. This activates the transcription of a series of cytokines and chemokines, which leads to inflammation [65]. The activation of NF-*κ*B requires posttranscriptional modifications such as methylation, acetylation, and phosphorylation. In this process, RelA/p65 is a subunit of NF-*κ*B, and its deacetylation effect needs special attention. Studies have reported that SIRT1 has a deacetylation effect on p65. SIRT1 inhibits the transcriptional activity of NF-*κ*B and the expression of proinflammatory cytokines, which can be achieved by deacetylation of p65 by overexpression or the activator resveratrol [66]. SIRT1 can reduce neuronal damage by inhibiting the activation of microglia. It may also reduce ischemic brain injury through anti-inflammatory effects [66]. Wei et al. found that Dishengzhu water decoction may prevent VD by downregulating the expression of NF-*κ*B in the hippocampus of VD mice [67]. Studies have shown that SIRT1 can deacetylate NF-*κ*B subunit RelA/p65, thereby inhibiting its transcriptional activity, reducing the production of inflammatory factors, and preventing damage caused by inflammation [68, 69]. The results of this experiment showed that after modeling, the levels of SIRT1 and I*κ*B*α* protein in the hippocampus of rats were significantly reduced, while the level of NF-*κ*B protein was significantly increased. After drug treatment, the level of SIRT1 and I*κ*B*α* protein in the hippocampus increased significantly, and the level of NF-*κ*B protein decreased significantly. This suggests that MBHD may have a certain ameliorating effect on neuronal inflammation in VD rats through the SIRT1/NF-*κ*B p65 inflammatory pathway.

In addition, the apoptosis signal pathway mediated by SIRT1 participates in the programmed cell death of neurons in VD rats and damages the function of neural units. Current research shows that cell necrosis and apoptosis coexist in cerebral infarction lesions after hypoxia-ischemia. The central area of ischemia mainly exists in the form of cell necrosis, and apoptosis is a major way of cell death in the ischemic penumbra around the infarct [70]. The functional area most closely related to learning and memory is the hippocampal CA1 area of the brain, which is very sensitive to hypoxia and ischemia [71]. Studies have found that a large amount of apoptosis and loss of hippocampal neurons in vascular dementia rats may be the pathological basis of vascular dementia [72]. The specific mechanism of apoptosis is currently not fully understood. It may be related to a variety of factors in apoptotic cells. The expression of apoptotic genes in apoptotic cells may directly lead to apoptosis. In the process of cell apoptosis, Bax, Bcl-2 gene family, and caspases family (including caspase-1, caspase-2, and caspase-3) are mostly studied. Numerous studies have proved that Bcl-2 and Bax are the two main genes in the apoptosis family that regulate cell apoptosis, and the abnormal expression of Bcl-2 and Bax proteins is closely related to cell apoptosis [73]. The expression level of Bcl-2 and Bax is directly related to the regulation of apoptosis and the severity of apoptosis. The increase of Bcl-2 inhibits cell apoptosis, while the increase of Bax promotes cell apoptosis. Bcl2 can be used as an endogenous neuroprotective substance. The high expression of Bcl-2 can prevent cell death caused by a variety of apoptosis factors and prolong cell life. Bax can act through its own homodimer to promote cell apoptosis. Bax is a downstream factor of SIRT1. Resveratrol can act on Ku70 mediated by SIRT1. SIRT1 is not acetylated and promotes its binding with Bax to play an antiapoptotic effect [74]. It is reported in the literature that Polygonum multiflorum inhibits cell apoptosis by regulating the expression of Bcl-2 and Bax protein, thereby improving the learning and cognitive abilities of rats with vascular cognitive dysfunction [75]. After cerebral ischemia and reperfusion, caspase-3 is activated to participate in the regulation of neuronal apoptosis, and it plays a very important role in participating in internal and external apoptosis pathways [76, 77]. SIRT1 can exert its antiapoptotic effect by increasing the expression of the antiapoptotic gene Bcl-2 in neurons and then reducing the expression of the proapoptotic gene caspase-3 [78]. In this experiment, the expression of SIRT1, Bax, Bcl-2, and caspase-3 in rat hippocampal neurons was detected by immunohistochemistry, QT-PCR, and Western blot, and the results showed that the expressions of SIRT1 and Bcl-2 in the hippocampus of vascular dementia model rats were significantly decreased, while Bax and caspase-3 were significantly increased. After drug treatment, both SIRT1 and Bcl-2 increased significantly, while Bax and caspase-3 decreased significantly, consistent with literature reports. This suggests that MBHD can protect rat hippocampal neurons by upregulating SIRT1 and Bcl-2 and downregulating Bax and caspase-3 to inhibit apoptosis.

In summary, MBHD may protect hippocampal neurons by regulating the SIRT1/NF-*κ*B p65 signaling pathway and then improve the learning and memory abilities of VD rats.

## 4. Conclusion

The active compounds of MBHD interact with multiple targets and multiple pathways in a synergistic manner, and have important therapeutic effects on VD mainly by balancing oxidative stress/anti-inflammatory and antiapoptotic, enhancing metabolism, and enhancing the immune system.

## Figures and Tables

**Table 1:** The primer.

Primer	Direction	Sequence (5′ to 3′)
SIRT1	Forward	AGGCAGACAATTTAATGGGGTGAA
SIRT1	Reverse	GAAGTCCACAGCAAGGCGAG
NF-*κ*B	Forward	TCCAGGTCATAGAGAGGCTCA
NF-*κ*B	Reverse	CCGTGGAGTACGACAACATCT
I*κ*B*α*	Forward	CTCAAGAAGGAGCGGTTGGT
I*κ*B*α*	Reverse	CCAAGTGCAGGAACGAGTCT
GAPDH	Forward	AGTGCCAGCCTCGTCTCATA
GAPDH	Reverse	GACTGTGCCGTTGAACTTGC
Bax	Forward	AGGACGCATCCACCAAGAAG
Bax	Reverse	CAGTTGAAGTTGCCGTCTGC
Bcl-2	Forward	GGATGACTTCTCTCGTCGCT
Bcl-2	Reverse	GACATCTCCCTGTTGACGCT
Caspase-3	Forward	CGCTGGACTGCGGTATTGAGA
Caspase-3	Reverse	TAACCGGGTGCGGTAGAGTA
GAPDH	Forward	AGTGCCAGCCTCGTCTCATA
GAPDH	Reverse	GACTGTGCCGTTGAACTTGC

**Table 2:** Linear relationship of standard curve.

Injection volume (ng)	18.8	37.6	75.3	112.9	150.6	188.2
Peak area	67.2	134.0	278.0	422.3	565.1	709.2

**Table 3:** Precision test.

Sample	1	2	3	4	5	*x*	RSD (%)
Peak area	2097	2077	2148	2054	2166	2108	2.24

**Table 4:** Repeatability test.

Sample	1	2	3	4	5	*x*	RSD (%)
Content (mg/100 ml)	2.1592	2.196	2.073	2.131	2.090	2.130	2.35

**Table 5:** Recovery test.

Sample	Content (mg)	Added content (mg)	Measured content (mg)	Recovery rate *X* (%)	*x* (%)	RSD (%)
1	0.1457	0.1506	0.2870	96.94	97.15	1.87
2	0.1447	0.1506	0.2773.	93.90		
3	0.1875	0.1882	0.3696	98.38		
4	0.1848	0.1882	0.3695	99,06		
5	0.2225	0.2258	0.4387	97.86		
6	0.2185	0.2258	0.4298	96.74		

**Table 6:** Recovery test.

Sample	Results of two parallel injections		*x* ± *s*	RSD (%)
1	18.07	17.57	17.51 ± 0.40	2.34
2	17.28	17.50		
3	16.83	17.29		

**Table 7:** Clusters of MBHD-VD PPI network.

Cluster	Score	Nodes	Edges	Targets and genes
1	52.567	68	1761	PIK3CD, PIK3CG, PIK3CA, IL1B, PLA2G1B, ERBB2, NGF, BDNF, MAPK14, MAPK9, CASP3, CAT, CSF2, GFAP, CALM1, TIMP1, EGFR, ESR1, CALM3, NOS3, STAT3, VEGFA, TNF, HSP90AA1, CALM2, ACE, IGF1, APOE, APP, TLR9, SERPINE1, ICAM1, MMP9, FGF2, NR3C1, TGFB1, REL, RELA, GAPDH, CREB1, JUN, AKT1, CASP9, BCL2, BCL2L1, PTGS2, MAPK8, MAPK3, CYCS, AR, KDR, FLT1, F2, CXCR4, FAS, IL6, NOS2, MAPK1, PLG, CASP8, PPARG, MT3, SRC, CD40, TF, STAT1, TNFRSF1A, MMP2
2	8	29	112	ESR2, PRKCG, GRM5, FPR2, PLCB1, MAPT, GSK3A, PTAFR, FGFR1, SREBF1, GRM1, SOD1, LRRK2, CRH, FLT4, AVP, ITIH4, CHRM1, CDK5, NPY, HCK, HTR2A, ADRA1B, ADRA1A, S100B, ADRA1D, CHRM3, LGALS3, CHRM5
3	6	20	57	CETP, APLP2, SLC6A3, IDE, OGT, SREBF2, BACE1, MBNL1, ECE1, AATF, APLP1, ABCA1, CSTB, NR1H3, NR1H2, HMGCR, TTR, LRP1, PTPRF, PRNP
4	5.769	27	75	FGF1, AGT, ERBB3, JUND, AKT2, NOS1, IL5, MET, MBP, LDLR, SYK, GSK3B, MCL1, PLAU, MMP3, MMP1, BCR, LYN, LCK, FYN, ADIPOQ, CDK4, CDK1, VWF, AGTR1, MMP13, HMOX1
5	5.667	25	68	CHRM4, PRKCB, CHRM2, ERN1, ADORA1, SYP, SNCA, ACTC1, GRIN2B, CNR2, CNR1, HTR1B, LPL, HTR1A, APOA1, MAP2, PPP3CA, BLK, HSP90B1, GAL, FGR, F7, YES1, APOB, PRKCD
6	4.8	6	12	ALDH1A2, CRYZ, ALDH2, ALDH1A3, CBR3, CBR1
7	4	7	12	VIP, SLC18A3, UBQLN2, ACHE, DNM1, PICALM, UBB
8	4	5	8	AKR1A1, AKR1B10, ALDH1L1, AKR1B15, ALDH1L2
9	3.667	7	11	AOX1, MAOA, ALDH1B1, ALDH1A1, RDH8, AKR1B1, HSD11B1L
10	3.5	13	21	TOP2A, A2M, SERPINA3, FGFR3, DLG4, HSP90AB1, CLU, GRIN2A, PSEN1, GRIN1, APOH, ELANE, CTSB
11	3.2	6	8	PRKCA, MAPK11, ADAM17, ERBB4, GDNF, NGFR
12	3	3	3	GLI3, GLI2, GLI1
13	3	5	6	DLST, MT-CO1, DHFRL1, SDHB, DHFR
14	3	3	3	LBR, CYP46A1, CYP51A1

**Table 8:** The binding energy of molecules (kcal/mol).

Components	BAX	BCL2	CASP3	RELA	SIRT1
64474-51-7	-4.71	-4	-5.89	-6.93	-3
Astragaloside IV	-1.02	-2.01	-4.2	-3.66	-3
Cycloartenol	-5.44	-5.74	-6.96	-7.38	-4.07
Ferulic acid	-3.17	-2.43	-5.82	-3.47	-3.33
Formononetin	-5.16	-4.82	-6.2	-7.21	-4.13
Hyrcanoside	-2.13	-4.07	-4.51	-4.33	-1.32
Kaempferol	-3.67	-4.41	-5.18	-6	-3.08
Ligustilide	-4.05	-4.04	-4.84	-5.03	-3.28
Liquiritigenin	-4.47	-5.63	-5.8	-6.65	-3.53
Mairin	-5.08	-6.05	-7	-6.69	-4.65
Mandenol	-1.35	-1.39	-3.12	-2.49	-0.36
Quercetin	-4.16	-5.18	-4.91	-5.87	-3.22
Senkyunone	-3.65	-3.66	-5.2	-4.73	-2.03
Wallichilide	-4.17	-3.49	-5.46	-5.78	-2.36
Xanthinin	-4.4	-4.67	-5.32	-6.33	-4.01

## Data Availability

The data used to support the findings of this study are included within the article and the supplementary information files.

## Supplementary Materials

Supplementary 1 Figure S1: standard curve of ferulic acid.

Supplementary 2 Figure S2: HPLC results (A: ferulic acid standard; B: negative control solution; C: MBHD sample).

Supplementary 3 Table S1: compound targets for MBHD.

Supplementary 4 Table S2: VD genes.

Supplementary 5 Table S3: biological processes of MBHD-VD PPI network.

Supplementary 6 Table S4: signaling pathway of MBHD-VD PPI network.

Supplementary 7 Table S5: enrichment analysis of differentially expressed protein PPI network.
