# Supplementary figures and images for: Exploring the Oxidative Stress Mechanism of Buyang Huanwu Decoction in Intervention of Vascular Dementia Based on Systems Biology Strategy
Source: Oxid Med Cell Longev. 2021 Mar 3;2021:8879060. doi: 10.1155/2021/8879060 (PMC7953864; doi:10.1155/2021/8879060)

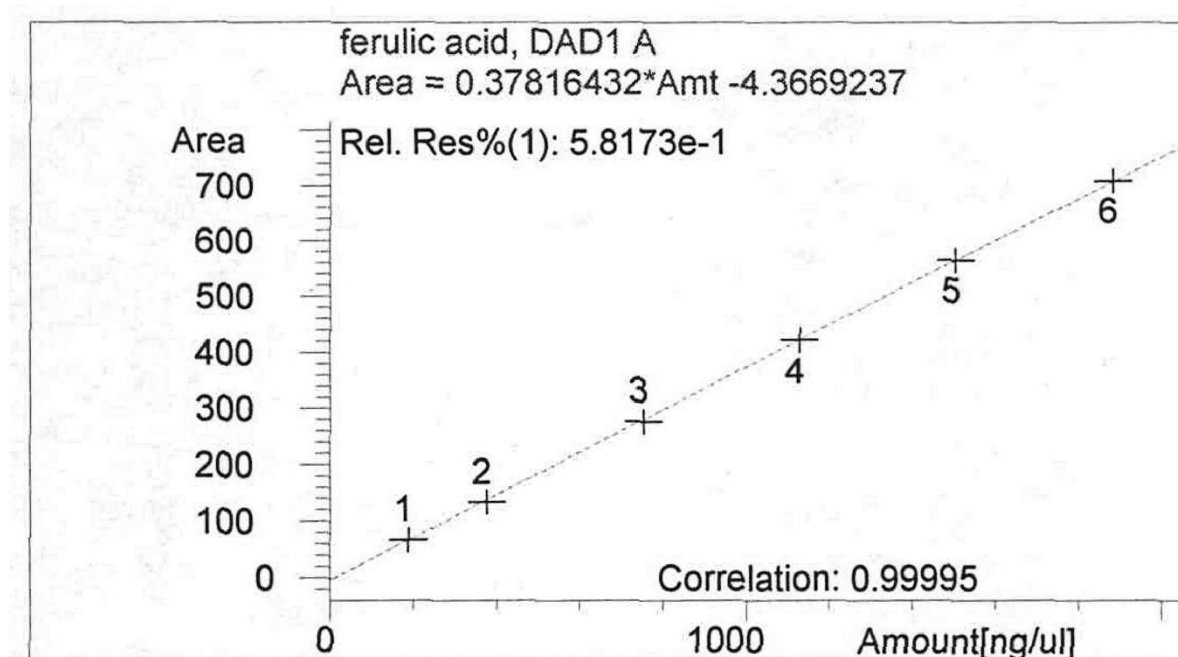

Figure S1 Standard curve of ferulic acid

Supplement: Supplementary 1 — Figure S1: standard curve of ferulic acid. [file 8879060.f1.pdf]

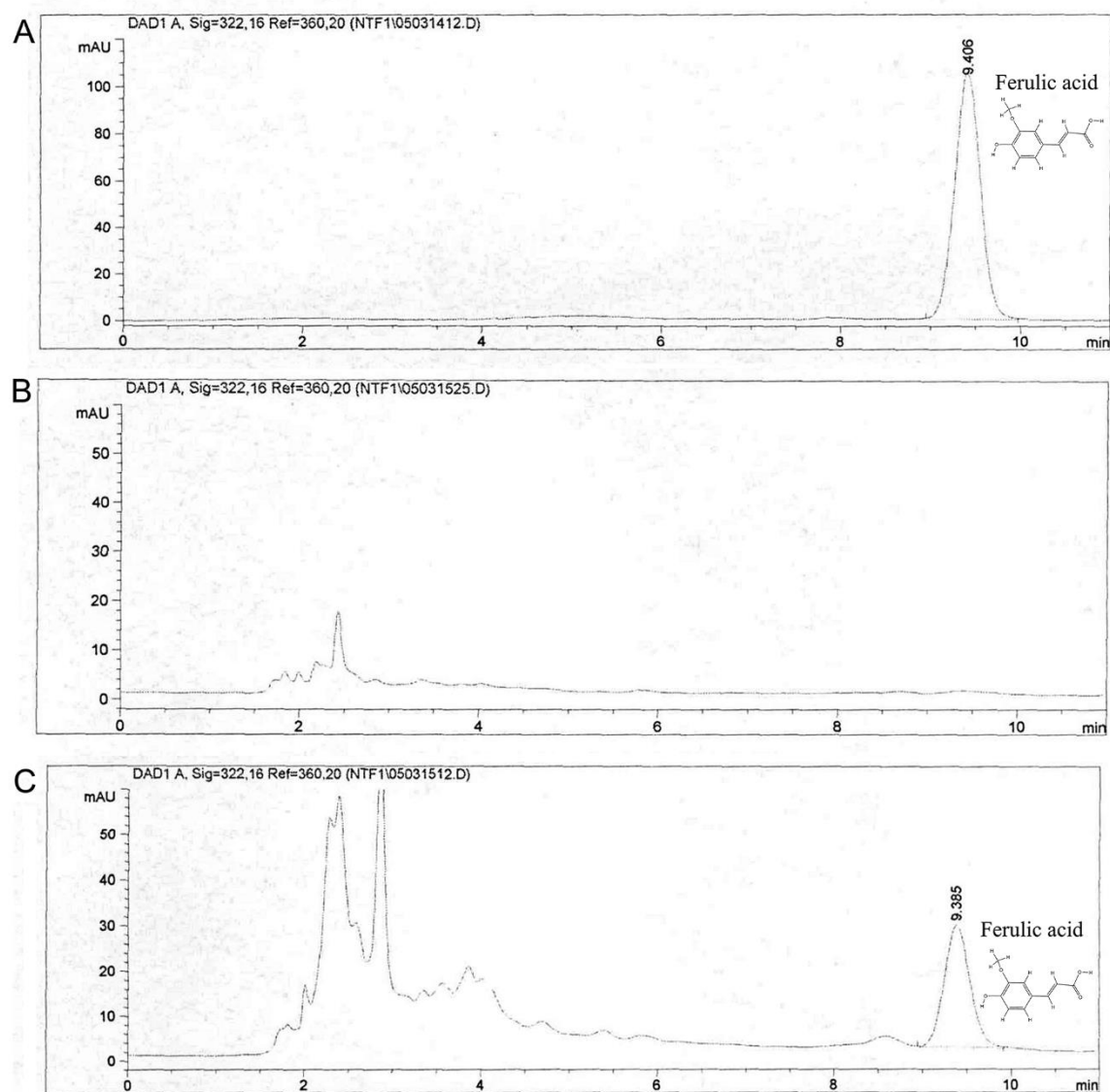

Figure S2 HPLC results (A: Ferulic acid standard; B: Negative control solution; C: MBHD sample)

Supplement: Supplementary 2 — Figure S2: HPLC results (A: ferulic acid standard; B: negative control solution; C: MBHD sample). [file 8879060.f2.pdf]
