# Supplementary material for: Exploring the Oxidative Stress Mechanism of Buyang Huanwu Decoction in Intervention of Vascular Dementia Based on Systems Biology Strategy
Source: Oxid Med Cell Longev. 2021 Mar 3;2021:8879060. doi: 10.1155/2021/8879060 (PMC7953864; doi:10.1155/2021/8879060)
Supplement: Supplementary 3 — Table S1: compound targets for MBHD. [file 8879060.f3.pdf]

Table S1 Compound targets of MBHD

Compound	Potential Targets
1,7-Dihydroxy-3,9-dir	MAPT
1,7-Dihydroxy-3,9-dir	ESR1
1,7-Dihydroxy-3,9-dir	ESR2
1,7-Dihydroxy-3,9-dir	PLA2G1B
1,7-Dihydroxy-3,9-dir	PTPN1
1,7-Dihydroxy-3,9-dir	PTPN2
1,7-Dihydroxy-3,9-dir	ADORA3
1,7-Dihydroxy-3,9-dir	PTGS1
1,7-Dihydroxy-3,9-dir	PTGS2
1,7-Dihydroxy-3,9-dir	MBNL1
1,7-Dihydroxy-3,9-dir	MBNL2
1,7-Dihydroxy-3,9-dir	MBNL3
1,7-Dihydroxy-3,9-dir	ADORA1
1,7-Dihydroxy-3,9-dir	ADORA2A
1,7-Dihydroxy-3,9-dir	DYRK1A
3,6′-Disinapoyl sucr	PRKCG
3,6′-Disinapoyl sucr	PRKCB
3,6′-Disinapoyl sucr	PRKCA
3,6′-Disinapoyl sucr	PRKCQ
3,6′-Disinapoyl sucr	PRKCD
3,6′-Disinapoyl sucr	MMP2
3,6′-Disinapoyl sucr	MMP9
3,6′-Disinapoyl sucr	MMP12
3,6′-Disinapoyl sucr	MMP13
3,6′-Disinapoyl sucr	MMP1
3,6′-Disinapoyl sucr	MMP3
3,6′-Disinapoyl sucr	MMP10
3,6′-Disinapoyl sucr	MMP27
3,6′-Disinapoyl sucr	AKR1B10
3,6′-Disinapoyl sucr	AKR1B1
3,9-di-O-methylnissol	PTPN1
3,9-di-O-methylnissol	PTPN2
3,9-di-O-methylnissol	FYN
3,9-di-O-methylnissol	YES1
3,9-di-O-methylnissol	FGR
3,9-di-O-methylnissol	SRC
3,9-di-O-methylnissol	FRK
3,9-di-O-methylnissol	CRYZ
3,9-di-O-methylnissol	ALOX15B
3,9-di-O-methylnissol	ALOX5
3,9-di-O-methylnissol	ALOX15
3,9-di-O-methylnissol	ALOXE3
3,9-di-O-methylnissol	CYP19A1
3,9-di-O-methylnissol	ALOX12
3,9-di-O-methylnissol	ALOX12B
4-Guanidino-1-butano	NOS3

4-Guanidino-1-butano	NOS1
4-Guanidino-1-butano	NOS2
4-Guanidino-1-butano	DHPS
4-Guanidino-1-butano	CA1
4-Guanidino-1-butano	CA2
4-Guanidino-1-butano	CA5A
4-Guanidino-1-butano	CA9
4-Guanidino-1-butano	CA5B
4-Guanidino-1-butano	CA3
4-Guanidino-1-butano	CA7
4-Guanidino-1-butano	CA13
4-Guanidino-1-butano	CXCR4
4-Guanidino-1-butano	TDP1
4-Guanidino-1-butano	F2
64474-51-7	ALOX15B
64474-51-7	ALOX5
64474-51-7	ALOX15
64474-51-7	P18054
64474-51-7	ALOXE3
64474-51-7	FLT1
64474-51-7	FLT4
64474-51-7	KDR
64474-51-7	MAPT
64474-51-7	MET
64474-51-7	CYP19A1
64474-51-7	FYN
64474-51-7	YES1
64474-51-7	FGR
64474-51-7	ALOX12
64474-51-7	ALOX12B
64997-52-0	AR
64997-52-0	TDP1
64997-52-0	CYP19A1
64997-52-0	HMGCR
64997-52-0	CYP51A1
64997-52-0	NR1H2
64997-52-0	NR1H3
64997-52-0	LDLR
64997-52-0	VLDLR
64997-52-0	LRP8
64997-52-0	SREBF2
64997-52-0	SREBF1
64997-52-0	ESR1
64997-52-0	ESR2
64997-52-0	CYP17A1
7-O-methylisomucron	ALOX15B
7-O-methylisomucron	ALOX5
7-O-methylisomucron	ALOX15

7-O-methylisomucron	ALOXE3
7-O-methylisomucron	FYN
7-O-methylisomucron	YES1
7-O-methylisomucron	FGR
7-O-methylisomucron	SRC
7-O-methylisomucron	FLT1
7-O-methylisomucron	FLT4
7-O-methylisomucron	KDR
7-O-methylisomucron	FRK
7-O-methylisomucron	MET
7-O-methylisomucron	ALOX12
7-O-methylisomucron	ALOX12B
73340-41-7	CRYZ
73340-41-7	PTPN1
73340-41-7	PTPN2
73340-41-7	FLT1
73340-41-7	FLT4
73340-41-7	KDR
73340-41-7	CYP19A1
73340-41-7	MET
73340-41-7	FGFR1
73340-41-7	FGFR2
73340-41-7	FGFR4
73340-41-7	FGFR3
73340-41-7	ALOX15B
73340-41-7	ALOX5
73340-41-7	ALOX15
8-Isopentenyl-kaempf	PDE5A
8-Isopentenyl-kaempf	PDE11A
8-Isopentenyl-kaempf	CYP19A1
8-Isopentenyl-kaempf	AKT1
8-Isopentenyl-kaempf	AKT2
8-Isopentenyl-kaempf	AKT3
8-Isopentenyl-kaempf	PTPN1
8-Isopentenyl-kaempf	PTPN2
8-Isopentenyl-kaempf	ABCG2
8-Isopentenyl-kaempf	BACE1
8-Isopentenyl-kaempf	BACE2
8-Isopentenyl-kaempf	ALOX5
8-Isopentenyl-kaempf	ALOX15
8-Isopentenyl-kaempf	ALOX12
8-Isopentenyl-kaempf	ALOX15B
alpha-Asarone	CYP19A1
alpha-Asarone	NQO1
alpha-Asarone	NQO2
alpha-Asarone	MAPT
alpha-Asarone	GFER
alpha-Asarone	TUBB1

alpha-Asarone	TUBB8
alpha-Asarone	MC1R
alpha-Asarone	PTGS1
alpha-Asarone	PTGS2
alpha-Asarone	ESR1
alpha-Asarone	ESR2
alpha-Asarone	MAOA
alpha-Asarone	MAOB
Astragaloside IV	FGF1
Astragaloside IV	FGF2
Astragaloside IV	VEGFA
Astragaloside IV	HPSE
Astragaloside IV	HPSE2
Astragaloside IV	CHRM1
Astragaloside IV	CHRM2
Astragaloside IV	CHRM4
Astragaloside IV	CHRM5
Astragaloside IV	CHRM3
Astragaloside IV	ADRA1D
Astragaloside IV	ADRA1A
Astragaloside IV	ADRA1B
Astragaloside IV	CDK1
Astragaloside IV	CDK4
beta-Asarone	MAPT
beta-Asarone	CYP19A1
beta-Asarone	NQO1
beta-Asarone	NQO2
beta-Asarone	TUBB1
beta-Asarone	TUBB8
beta-Asarone	GFER
beta-Asarone	PTGS1
beta-Asarone	PTGS2
beta-Asarone	MAOA
beta-Asarone	MAOB
beta-Asarone	MC1R
beta-Asarone	ESR1
beta-Asarone	ESR2
beta-Sitosterol	TDP1
beta-Sitosterol	CYP17A1
beta-Sitosterol	NR1H2
beta-Sitosterol	NR1H3
beta-Sitosterol	HMGCR
beta-Sitosterol	SREBF2
beta-Sitosterol	SREBF1
beta-Sitosterol	AR
beta-Sitosterol	LDLR
beta-Sitosterol	VLDLR
beta-Sitosterol	LRP8

beta-Sitosterol	SLC6A4
beta-Sitosterol	BCHE
beta-Sitosterol	ACHE
beta-Sitosterol	SLC6A2
Bifendate	TDP1
Bifendate	ALOX5
Bifendate	ALOX15B
Bifendate	ALOX15
Bifendate	ALOXE3
Bifendate	MAPT
Bifendate	ADORA1
Bifendate	F10
Bifendate	F9
Bifendate	F7
Bifendate	HSP90AA1
Bifendate	HSP90AB1
Bifendate	HSP90B1
Bifendate	ALOX12
Bifendate	ALOX12B
Butylidenephthalide	MBNL1
Butylidenephthalide	MBNL2
Butylidenephthalide	MBNL3
Butylidenephthalide	MAPT
Butylidenephthalide	CYP19A1
Butylidenephthalide	LCK
Butylidenephthalide	LYN
Butylidenephthalide	HCK
Butylidenephthalide	BLK
Butylidenephthalide	PLAU
Butylidenephthalide	HGFAC
Butylidenephthalide	ESR1
Butylidenephthalide	ESR2
Butylidenephthalide	FLT3
Butylidenephthalide	STAT3
Calycosin 7-O-glucosi	ALDH2
Calycosin 7-O-glucosi	ALDH1L1
Calycosin 7-O-glucosi	ALDH1A2
Calycosin 7-O-glucosi	ALDH1A1
Calycosin 7-O-glucosi	ALDH1B1
Calycosin 7-O-glucosi	ALDH1A3
Calycosin 7-O-glucosi	ALDH1L2
Calycosin 7-O-glucosi	MBNL1
Calycosin 7-O-glucosi	MBNL2
Calycosin 7-O-glucosi	MBNL3
Calycosin 7-O-glucosi	ADORA1
Calycosin 7-O-glucosi	TDP1
Calycosin 7-O-glucosi	AKR1B1
Calycosin 7-O-glucosi	AKR1B15

Calycosin	MAPT
Calycosin	HSD17B2
Calycosin	HSD11B2
Calycosin	ESR1
Calycosin	ESR2
Calycosin	TDP1
Calycosin	ALOX5
Calycosin	ALOX15
Calycosin	ALOX15B
Calycosin	ALOXE3
Calycosin	ADORA1
Calycosin	CYP19A1
Calycosin	EGFR
Calycosin	ALOX12
Calycosin	ALOX12B
Cholesteryl ferulate	POLB
Cholesteryl ferulate	ELANE
Cholesteryl ferulate	AZU1
Cholesteryl ferulate	PRTN3
Cholesteryl ferulate	MMP1
Cholesteryl ferulate	MMP2
Cholesteryl ferulate	MMP3
Cholesteryl ferulate	MMP9
Cholesteryl ferulate	MMP12
Cholesteryl ferulate	MMP13
Cholesteryl ferulate	MMP10
Cholesteryl ferulate	MMP27
Cholesteryl ferulate	ALOX5
Cholesteryl ferulate	ALOX15
Cholesteryl ferulate	ALOX12
Cycloartenol	NR1H2
Cycloartenol	NR1H3
Cycloartenol	LDLR
Cycloartenol	VLDLR
Cycloartenol	LRP8
Cycloartenol	TDP1
Cycloartenol	HMGCR
Cycloartenol	CYP51A1
Cycloartenol	CYP19A1
Cycloartenol	SLC6A2
Cycloartenol	SLC6A4
Cycloartenol	SLC6A3
Cycloartenol	SLC6A9
Cycloartenol	SLC6A7
Cycloartenol	SLC6A14
Diop	MAPT
Diop	PRKCG
Diop	PRKCB

Diop	PRKCA
Diop	PRKCD
Diop	PRKCQ
Diop	EPHX2
Diop	PTPN2
Diop	PTPN1
Diop	AR
Diop	MGLL
Diop	CNR1
Diop	CNR2
Diop	BCHE
Diop	ACHE
Eudesmin	PTAFR
Eudesmin	ALOX5
Eudesmin	ALOX15
Eudesmin	ALOX12
Eudesmin	ALOX15B
Eudesmin	ALOX12B
Eudesmin	ALOXE3
Eudesmin	MAPT
Eudesmin	MAPK8
Eudesmin	MAPK9
Eudesmin	MAPK10
Eudesmin	MAPK14
Eudesmin	MAPK11
Eudesmin	PTGS1
Eudesmin	PTGS2
Ferulic Acid	CA12
Ferulic Acid	CA1
Ferulic Acid	CA2
Ferulic Acid	CA3
Ferulic Acid	CA6
Ferulic Acid	CA5A
Ferulic Acid	CA7
Ferulic Acid	CA9
Ferulic Acid	CA13
Ferulic Acid	CA14
Ferulic Acid	CA5B
Ferulic Acid	TDP1
Ferulic Acid	AKR1B10
Ferulic Acid	AKR1B1
Ferulic Acid	AKR1B15
Formononetin	MAPT
Formononetin	HSD17B2
Formononetin	HSD11B2
Formononetin	CYP19A1
Formononetin	CBR1
Formononetin	CBR3

Formononetin	ESR1
Formononetin	ESR2
Formononetin	EGFR
Formononetin	ERBB2
Formononetin	ERBB3
Formononetin	ERBB4
Formononetin	TDP1
Formononetin	HSD17B1
Formononetin	RDH8
Guanosine	NOS3
Guanosine	NOS1
Guanosine	NOS2
Guanosine	DHFR
Guanosine	DHFRL1
Guanosine	ADK
Guanosine	MAPT
Guanosine	GRM5
Guanosine	APP
Guanosine	BACE1
Guanosine	BACE2
Guanosine	APLP2
Guanosine	PIK3CA
Guanosine	PIK3CG
Guanosine	PIK3CD
Hederagenin	PTPN2
Hederagenin	PTPN1
Hederagenin	AKR1B10
Hederagenin	AKR1B15
Hederagenin	AKR1A1
Hederagenin	AKR1B1
Hederagenin	PLA2G1B
Hederagenin	POLB
Hederagenin	PTPRF
Hederagenin	ACP1
Hederagenin	HSD11B1
Hederagenin	PTPRD
Hederagenin	PTPRS
Hederagenin	HSD11B1L
Hederagenin	AKR1E2
Hyrcanoside	MAPT
Hyrcanoside	STAT3
Hyrcanoside	STAT1
Hyrcanoside	STAT2
Hyrcanoside	STAT4
Hyrcanoside	ADORA3
Hyrcanoside	PRKCG
Hyrcanoside	PRKCB
Hyrcanoside	PRKCA

Hyrcanoside	PRKCQ
Hyrcanoside	PRKCD
Hyrcanoside	SLC6A9
Hyrcanoside	SLC6A7
Hyrcanoside	SLC6A14
Hyrcanoside	SLC6A5
Isoflavanone	ALOX15B
Isoflavanone	ALOX5
Isoflavanone	ALOX15
Isoflavanone	P18054
Isoflavanone	ALOXE3
Isoflavanone	CYP19A1
Isoflavanone	FLT1
Isoflavanone	FLT4
Isoflavanone	KDR
Isoflavanone	ESR1
Isoflavanone	ESR2
Isoflavanone	MET
Isoflavanone	MAPT
Isoflavanone	TDP1
Isorhamnetin	CYP1A2
Isorhamnetin	XDH
Isorhamnetin	CYP1B1
Isorhamnetin	CYP1A1
Isorhamnetin	AOX1
Isorhamnetin	MAPT
Isorhamnetin	AKR1B1
Isorhamnetin	AKR1B15
Isorhamnetin	AKR1B10
Isorhamnetin	NOX4
Isorhamnetin	AKR1A1
Isorhamnetin	ABCC1
Isorhamnetin	ABCC3
Isorhamnetin	ABCC2
Isorhamnetin	AKR1E2
Jaranol	ADORA1
Jaranol	ADORA3
Jaranol	DYRK1A
Jaranol	ABCG2
Jaranol	MAPT
Jaranol	PLG
Jaranol	NOX4
Jaranol	LPA
Jaranol	ADORA2A
Jaranol	AKR1B10
Jaranol	AKR1B1
Jaranol	AKR1B15
Jaranol	MCL1

Jaranol	ALOX5
Jaranol	ALOX15
Kaempferol	CYP1A2
Kaempferol	ABCB1
Kaempferol	ALOX5
Kaempferol	HSD17B1
Kaempferol	AKR1B1
Kaempferol	ALOX15
Kaempferol	ABCC1
Kaempferol	AHR
Kaempferol	HSD17B2
Kaempferol	XDH
Kaempferol	DYRK1A
Kaempferol	CYP1B1
Kaempferol	NOX4
Kaempferol	TDP1
Kaempferol	ALOX12
Ligustilide	ALOX5
Ligustilide	ALOX15
Ligustilide	ALOX15B
Ligustilide	ALOXE3
Ligustilide	SLC6A2
Ligustilide	SLC6A4
Ligustilide	SLC6A3
Ligustilide	SLC6A9
Ligustilide	SLC6A7
Ligustilide	SLC6A14
Ligustilide	SLC6A5
Ligustilide	TDP1
Ligustilide	ALOX12
Ligustilide	ALOX12B
Ligustilide	CYP17A1
Liquiritigenin	MAPT
Liquiritigenin	MAOA
Liquiritigenin	MAOB
Liquiritigenin	CYP19A1
Liquiritigenin	TDP1
Liquiritigenin	HSD17B1
Liquiritigenin	RDH8
Liquiritigenin	ABCG2
Liquiritigenin	CYP1A2
Liquiritigenin	CBR1
Liquiritigenin	CYP1B1
Liquiritigenin	CYP1A1
Liquiritigenin	CBR3
Liquiritigenin	ESR1
Liquiritigenin	ESR2
Mairin	AKR1B10

Mairin	POLB
Mairin	AKR1B1
Mairin	AKR1B15
Mairin	AKR1A1
Mairin	UBA2
Mairin	SAE1
Mairin	MAPT
Mairin	HSD11B1
Mairin	HSD11B1L
Mairin	NR1H4
Mairin	CDC25A
Mairin	CDC25B
Mairin	TOP2A
Mairin	TOP2B
Mairin	AKR1E2
Mandenol	FAAH
Mandenol	CNR1
Mandenol	CNR2
Mandenol	TDP1
Mandenol	ALOX5
Mandenol	ALOX15
Mandenol	ALOX15B
Mandenol	P18054
Mandenol	ALOXE3
Mandenol	PTPN2
Mandenol	PTPN1
Mandenol	PPARG
Mandenol	PPARD
Mandenol	ALOX12B
Mandenol	ALOX12
Mandenol	CYP17A1
Myricanone	TDP1
Myricanone	CNR1
Myricanone	CNR2
Myricanone	ALOX15
Myricanone	ALOX12
Myricanone	ALOX5
Myricanone	ALOX15B
Myricanone	ALOX12B
Myricanone	ALOXE3
Myricanone	HTR1A
Myricanone	HTR1B
Myricanone	MAPT
Myricanone	HSD17B1
Myricanone	RDH8
Myricanone	MBNL1
Ononin	ALDH2
Ononin	ALDH1L1

Ononin	ALDH1A2
Ononin	ALDH1A1
Ononin	ALDH1B1
Ononin	ALDH1A3
Ononin	ALDH1L2
Ononin	MBNL1
Ononin	MBNL2
Ononin	MBNL3
Ononin	IL5
Ononin	CA12
Ononin	CA1
Ononin	CA2
Perlolyrine	GRM5
Perlolyrine	GRM1
Perlolyrine	MAPT
Perlolyrine	PDE4A
Perlolyrine	PDE4B
Perlolyrine	PDE4C
Perlolyrine	PDE4D
Perlolyrine	ESR1
Perlolyrine	ESR2
Perlolyrine	EGFR
Perlolyrine	ERBB2
Perlolyrine	ERBB3
Perlolyrine	ERBB4
Perlolyrine	ADK
Perlolyrine	ADORA2A
Polygalasaponin XXV	F2
Polygalasaponin XXV	GLI1
Polygalasaponin XXV	GLI2
Polygalasaponin XXV	GLI3
Polygalasaponin XXV	MAPT
Polygalasaponin XXV	BCL2L1
Polygalasaponin XXV	BCL2
Polygalasaponin XXV	BCL2L2
Polygalasaponin XXV	PPP1CC
Polygalasaponin XXV	PPP1CA
Polygalasaponin XXV	PPP1CB
Polygalasaponin XXV	PTAFR
Polygalasaponin XXV	JUN
Polygalasaponin XXV	JUNB
Polygalasaponin XXV	JUND
Polygalaxanthone III	MAPT
Polygalaxanthone III	MBNL1
Polygalaxanthone III	MBNL2
Polygalaxanthone III	MBNL3
Polygalaxanthone III	TDP1
Polygalaxanthone III	XDH

Polygalaxanthone III	AOX1
Polygalaxanthone III	AKR1B10
Polygalaxanthone III	AKR1B1
Polygalaxanthone III	AKR1B15
Polygalaxanthone III	DYRK1A
Polygalaxanthone III	ADORA1
Polygalaxanthone III	CA12
Polygalaxanthone III	CA1
Polygalaxanthone III	CA2
Quercetin	CA12
Quercetin	EGFR
Quercetin	CA1
Quercetin	CA2
Quercetin	PLA2G1B
Quercetin	ERBB2
Quercetin	MPO
Quercetin	CYP1A2
Quercetin	CDK1
Quercetin	CA3
Quercetin	PRSS1
Quercetin	MMP2
Quercetin	MMP3
Quercetin	ALOX5
Quercetin	MAPT
Senegin III	PRKCQ
Senegin III	PRKCD
Senegin III	FDFT1
Senegin III	F2
Senegin III	PRKCG
Senegin III	PRKCB
Senegin III	PRKCA
Senegin III	MMP2
Senegin III	MMP9
Senegin III	MMP12
Senegin III	MMP13
Senegin III	MMP1
Senegin III	MMP3
Senegin III	MMP10
Senegin III	MMP27
senkyunolide I	CRYZ
senkyunolide I	PRKCG
senkyunolide I	PRKCB
senkyunolide I	PRKCA
senkyunolide I	PRKCQ
senkyunolide I	PRKCD
senkyunolide I	PTGS1
senkyunolide I	PTGS2
senkyunolide I	RELA

senkyunolide I	REL
senkyunolide I	MMP1
senkyunolide I	MMP3
senkyunolide I	MMP8
senkyunolide I	MMP12
senkyunolide I	MMP13
Senkyunone	ALOX5
Senkyunone	ALOX15
Senkyunone	ALOX15B
Senkyunone	ALOX12B
Senkyunone	ALOX12
Senkyunone	ALOXE3
Senkyunone	CYP19A1
Senkyunone	NR3C1
Senkyunone	NR3C2
Senkyunone	MAPT
Senkyunone	BCHE
Senkyunone	ACHE
Senkyunone	TDP1
Senkyunone	ESR1
Senkyunone	ESR2
Sibiricaxanthone A	MAPT
Sibiricaxanthone A	MBNL1
Sibiricaxanthone A	MBNL2
Sibiricaxanthone A	MBNL3
Sibiricaxanthone A	TDP1
Sibiricaxanthone A	DYRK1A
Sibiricaxanthone A	CA12
Sibiricaxanthone A	CA1
Sibiricaxanthone A	CA2
Sibiricaxanthone A	CA3
Sibiricaxanthone A	CA7
Sibiricaxanthone A	CA9
Sibiricaxanthone A	CA13
Sibiricaxanthone A	CA14
Sibiricaxanthone A	CA5B
Sitosterol	TDP1
Sitosterol	AR
Sitosterol	HMGCR
Sitosterol	CYP51A1
Sitosterol	NR1H2
Sitosterol	NR1H3
Sitosterol	CYP19A1
Sitosterol	CYP17A1
Sitosterol	LDLR
Sitosterol	VLDLR
Sitosterol	LRP8
Sitosterol	ESR1

Sitosterol	ESR2
Sitosterol	SLC6A2
Sitosterol	SLC6A4
Stigmasterol	AR
Stigmasterol	TDP1
Stigmasterol	CYP19A1
Stigmasterol	HMGCR
Stigmasterol	CYP51A1
Stigmasterol	NR1H2
Stigmasterol	NR1H3
Stigmasterol	LDLR
Stigmasterol	VLDLR
Stigmasterol	LRP8
Stigmasterol	CYP17A1
Stigmasterol	ESR1
Stigmasterol	ESR2
Stigmasterol	SLC6A2
Stigmasterol	SLC6A3
Tenuifolin	BCL2L1
Tenuifolin	BCL2
Tenuifolin	BCL2L2
Tenuifolin	STAT3
Tenuifolin	STAT1
Tenuifolin	STAT2
Tenuifolin	STAT4
Tenuifolin	F2
Tenuifolin	NR3C1
Tenuifolin	NR3C2
Tenuifolin	HSD11B1
Tenuifolin	HSD11B2
Tenuifolin	HSD11B1L
Tenuifolin	MMP2
Tenuifolin	MMP9
Tenuifoliose A	MMP1
Tenuifoliose A	PRKCG
Tenuifoliose A	PRKCB
Tenuifoliose A	MMP2
Tenuifoliose A	MMP3
Tenuifoliose A	MMP9
Tenuifoliose A	PRKCA
Tenuifoliose A	MMP12
Tenuifoliose A	MMP13
Tenuifoliose A	PRKCQ
Tenuifoliose A	PRKCD
Tenuifoliose A	MMP10
Tenuifoliose A	MMP27
Tenuifoliose A	ABCB1
Tenuifoliose A	ABCB11

Tenuifoliose H	ABCB1
Tenuifoliose H	ABCB11
Tenuifoliose H	ABCB4
Tenuifoliose H	ABCB5
Tenuifoliose H	SQLE
Tenuifoliose H	PTPN2
Tenuifoliose H	PTPN1
Tenuifoliose H	PRKCG
Tenuifoliose H	PRKCB
Tenuifoliose H	MMP2
Tenuifoliose H	MMP9
Tenuifoliose H	PRKCA
Tenuifoliose H	MMP12
Tenuifoliose H	MMP13
Tenuifoliose H	PRKCQ
Tenuifoliose I	ABCB1
Tenuifoliose I	ABCB11
Tenuifoliose I	ABCB4
Tenuifoliose I	ABCB5
Tenuifoliose I	PRKCG
Tenuifoliose I	PRKCB
Tenuifoliose I	MMP2
Tenuifoliose I	MMP9
Tenuifoliose I	PRKCA
Tenuifoliose I	MMP12
Tenuifoliose I	MMP13
Tenuifoliose I	PRKCQ
Tenuifoliose I	PRKCD
Tenuifoliose I	MMP1
Tenuifoliose I	MMP3
Tenuifoliside A	SQLE
Tenuifoliside A	ABCB1
Tenuifoliside A	ABCB11
Tenuifoliside A	ABCB4
Tenuifoliside A	ABCB5
Tenuifoliside A	LGALS9
Tenuifoliside A	LGALS3
Tenuifoliside A	LGALS9B
Tenuifoliside A	LGALS9C
Tenuifoliside A	MMP1
Tenuifoliside A	PRKCG
Tenuifoliside A	PRKCB
Tenuifoliside A	MMP2
Tenuifoliside A	MMP3
Tenuifoliside A	MMP9
Tenuifoliside B	PRKCG
Tenuifoliside B	PRKCB
Tenuifoliside B	PRKCA

Tenuifoliside B	PRKCQ
Tenuifoliside B	PRKCD
Tenuifoliside B	MMP1
Tenuifoliside B	MMP2
Tenuifoliside B	MMP3
Tenuifoliside B	MMP9
Tenuifoliside B	MMP12
Tenuifoliside B	MMP13
Tenuifoliside B	MMP10
Tenuifoliside B	MMP27
Tenuifoliside B	TDP1
Tenuifoliside B	ABCB1
Wallichilide	TDP1
Wallichilide	PPARG
Wallichilide	PPARD
Wallichilide	PPARA
Wallichilide	MAPT
Wallichilide	ADORA1
Wallichilide	JUN
Wallichilide	JUNB
Wallichilide	JUND
Wallichilide	CYP19A1
Wallichilide	PTAFR
Wallichilide	FNTA
Wallichilide	HSD11B1
Wallichilide	HSD11B1L
Wallichilide	DUSP3
Wallichilide	FNTB
Xanthinin	PTGS1
Xanthinin	PTGS2
Xanthinin	MAPT
Xanthinin	FNTA
Xanthinin	CYP19A1
Xanthinin	PRKCG
Xanthinin	PRKCB
Xanthinin	PRKCA
Xanthinin	PRKCQ
Xanthinin	PRKCD
Xanthinin	RELA
Xanthinin	REL
Xanthinin	TLR9
Xanthinin	FNTB
Xanthinin	PTPN2
