# Supplementary material for: Exploring the Oxidative Stress Mechanism of Buyang Huanwu Decoction in Intervention of Vascular Dementia Based on Systems Biology Strategy
Source: Oxid Med Cell Longev. 2021 Mar 3;2021:8879060. doi: 10.1155/2021/8879060 (PMC7953864; doi:10.1155/2021/8879060)
Supplement: Supplementary 4 — Table S2: VD genes. [file 8879060.f4.pdf]

Table S2 VD Gene

MAPT
APP
APOE
PSEN1
ACE
TNF
ACHE
BCHE
SORL1
RETREG1
MTHFR
CHAT
NOTCH3
VEGFA
PLAU
NOS3
BLMH
SERPINA3
MPO
A2M
HFE
APBB2
PAXIP1
MT-ND1
MIR34A
MIR146A
MIR106B
MIR29A
MIR107
MIR29B1
MIR328
MIR298
AD5
AD10
AD6
AD7
AD11
AD12
AD13
AD14
AD15
AD16
AD17
AD8
PON1
CST3
PRNP

CRH
MBP
SOD1
IL1A
AGER
GFAP
SLC6A4
CTSD
GRIN1
GRIN2A
CAT
CASP3
GRIN2B
CHRNA4
COX5A
SNCA
BDNF
ITM2B
NGF
PSEN2
GRN
SYP
LTA
SQSTM1
TARDBP
BACE1
SNCB
IL1B
MAOB
IDE
CYP46A1
HMOX1
LRP1
S100B
CHRNA7
SNCG
HTR2A
ADAM17
TNFRSF1A
TF
CD40
VLDLR
CLU
DNM1L
GAL
VIP
REG1A
MT3

MIR210
PPARG
CASP8
NTRK2
ADAM10
MAPK1
CDK5
UCHL1
PPP3CA
CSNK1D
GSK3B
CASP7
FAS
LPL
MME
LDLR
CTSB
TTR
SLC1A2
SLC1A3
ITPR1
CYCS
MAPK3
MAPK8
NQO1
DYRK1A
PTGS2
GDNF
ABCA1
GSK3A
CASP9
CASP2
CAPN1
NOS1
CREB1
NTRK1
APAF1
PLCB1
GAPDH
CAPN2
ITPR3
EIF2AK3
NCSTN
FPR2
MSR1
ERN1
GLRX
HSD17B10

CDK5R1
DPYSL2
DKK1
SEMA3A
EIF2AK2
TNFRSF21
PIN1
RTN4
NGFR
PSENEN
GRIN2C
CALM2
DHCR24
DLG4
SLC18A3
OLR1
UBB
ABCA7
GPC1
APH1B
APBB1
HTR6
RELN
CALM3
RCAN1
MAP2
DLST
TMED10
OGT
APH1A
APLP2
APBA2
LRP8
MARK4
COX4I1
CALCA
CALM1
MAP1B
PICALM
UBQLN1
KLK8
GAP43
RYR3
RTN3
NAE1
VSNL1
PADI2
BACE2

APLP1
APOC1
CIB1
PLD3
AATF
MARK1
DOCK3
CTNNA3
VPS26A
TFCP2
BPTF
APBA3
ANKS1B
COL25A1
CLSTN1
DBN1
NRGN
STMN2
SLC39A1
GPR3
PITRM1
APBA1
KCNIP3
MEOX2
CALML5
NDRG2
SLC30A6
FRMD4A
DNMBP
APBB3
SORCS3
TTBK1
GRK2
PCSK1N
CALHM1
DCHS2
EXOC3L2
PTPA
GSAP
TM2D1
MT-CO1
MT-ND2
MT-CO2
ATP5PD
STH
MALRD1
ASAH2B
MIAT

MIR181C
MIR26B
SOX2-OT
HAR1A
MIR320A
CDKN2B-AS1
MIR93
MIR128-1
MIR22
MIRLET7I
MIR197
BCYRN1
HAR1B
MIR15A
MIR363
MIR511
SNHG3
LINC01080
BACE1-AS
LRP1-AS
LINC01772
LINC01616
CHGA
SERPINI1
SST
HTRA1
ITIH4
IL6
SYK
DNMT1
SLC6A3
TBK1
VCP
LRRK2
GBA
NPC1
HNRNPA1
ARSA
PARK7
CSTB
HNRNPA2B1
TRPM7
FUS
CHMP2B
TYROBP
UBQLN2
TREM2
ACTC1

PLP1
SNCAIP
NPC2
ATP13A2
C9orf72
CHCHD10
NHLRC1
RECK
PRKN
TMEM106B
TGFB1
AGT
SREBF2
MMP9
ICAM1
PLA2G2A
ESR2
NPPB
SERPINE1
APOB
APOH
AGTR1
MSBP2
VWF
PTH
ADIPOQ
NEFL
TNFSF14
CBS
IGF1
DCX
FABP3
GSTO1
AMBP
KLK6
BCR
MMP1
MMP3
LBR
CP
ECE1
NPY
CHRM1
AOC3
CDKN2A-DT
ESR1
DNM1
APOA1

CLDN1
HTR1A
ATP1B1
CETP
AVP
ADORA2A
OCLN
TIMP1
SLC19A1
PON3
LCN2
CHRNB2
KLK1
CSF2
SAR1B
LIF
PON2
ANGPT4
TNFRSF19
RPGRIP1L
TSC22D3
PHLDB2
LOC110806262
SDHB
PDE4A
