# Supplementary material for: Exploring the Oxidative Stress Mechanism of Buyang Huanwu Decoction in Intervention of Vascular Dementia Based on Systems Biology Strategy
Source: Oxid Med Cell Longev. 2021 Mar 3;2021:8879060. doi: 10.1155/2021/8879060 (PMC7953864; doi:10.1155/2021/8879060)
Supplement: Supplementary 5 — Table S3: biological processes of MBHD-VD PPI network. [file 8879060.f5.pdf]

Table S3 Biological Processes of MBHD-VD PPI Network

Cluster	Term	Pathway	Count	%	Pvalue
	GO:0010628	positive regulation of gene expression	16	23.52941	8.88E-14
	GO:0043066	negative regulation of apoptotic proce	19	27.94118	1.23E-13
	GO:0045429	positive regulation of nitric oxide bios	9	13.23529	5.42E-12
	GO:0050999	regulation of nitric-oxide synthase act	8	11.76471	7.23E-12
	GO:0002576	platelet degranulation	11	16.17647	8.95E-12
	GO:0048661	positive regulation of smooth muscle	9	13.23529	9.06E-11
	GO:0051092	positive regulation of NF-kappaB tran	11	16.17647	1.17E-10
	GO:0006954	inflammatory response	15	22.05882	2.32E-10
	GO:0030168	platelet activation	9	13.23529	1.76E-08
	GO:0001525	angiogenesis	11	16.17647	1.79E-08
	GO:0051000	positive regulation of nitric-oxide syn	6	8.823529	2.17E-08
	GO:0014066	regulation of phosphatidylinositol 3-k	8	11.76471	2.47E-08
	GO:0032355	response to estradiol	8	11.76471	7.25E-08
	GO:0001666	response to hypoxia	9	13.23529	4.01E-07
	GO:0048010	vascular endothelial growth factor rec	7	10.29412	4.08E-07
1	GO:1901215	negative regulation of neuron death	6	8.823529	5.13E-07
	GO:0043536	positive regulation of blood vessel en	5	7.352941	8.58E-07
	GO:0043524	negative regulation of neuron apoptot	8	11.76471	9.29E-07
	GO:0035924	cellular response to vascular endothel	5	7.352941	1.94E-06
	GO:0034097	response to cytokine	6	8.823529	1.95E-06
	GO:0045766	positive regulation of angiogenesis	7	10.29412	6.43E-06
	GO:0030949	positive regulation of vascular endoth	4	5.882353	3.28E-05
	GO:0043123	positive regulation of I-kappaB kinase	7	10.29412	4.36E-05
	GO:0042346	positive regulation of NF-kappaB imp	4	5.882353	7.67E-05
	GO:0051402	neuron apoptotic process	4	5.882353	3.04E-04
	GO:0010507	negative regulation of autophagy	4	5.882353	3.95E-04
	GO:0006979	response to oxidative stress	5	7.352941	9.73E-04
	GO:0030593	neutrophil chemotaxis	4	5.882353	0.002322
	GO:0007179	transforming growth factor beta recep	4	5.882353	0.005919
	GO:0043542	endothelial cell migration	3	4.411765	0.00594
	GO:0045765	regulation of angiogenesis	3	4.411765	0.006768
	GO:0045907	positive regulation of vasoconstriction	6	20.68966	1.73E-09
	GO:0008284	positive regulation of cell proliferation	9	31.03448	6.32E-07
	GO:0008283	cell proliferation	8	27.58621	1.76E-06
	GO:0007268	chemical synaptic transmission	7	24.13793	2.32E-06
	GO:0007197	adenylate cyclase-inhibiting G-protein	3	10.34483	5.60E-05
2	GO:0007204	positive regulation of cytosolic calciu	5	17.24138	6.84E-05
	GO:0006939	smooth muscle contraction	3	10.34483	4.04E-04
	GO:0007186	G-protein coupled receptor signaling	8	27.58621	5.41E-04
	GO:0051966	regulation of synaptic transmission, gl	3	10.34483	6.64E-04
	GO:0007611	learning or memory	3	10.34483	0.001631
	GO:0007271	synaptic transmission, cholinergic	3	10.34483	0.001722

3	GO:0010745	negative regulation of macrophage de	4	20	3.49E-07
	GO:0010875	positive regulation of cholesterol efflu	4	20	4.44E-07
	GO:0045861	negative regulation of proteolysis	4	20	1.61E-06
	GO:0010887	negative regulation of cholesterol stor	3	15	1.81E-05
	GO:2000188	regulation of cholesterol homeostasis	3	15	4.35E-05
	GO:0042632	cholesterol homeostasis	4	20	4.90E-05
	GO:0042157	lipoprotein metabolic process	3	15	8.32E-04
	GO:0008203	cholesterol metabolic process	3	15	0.002643
4	GO:0018108	peptidyl-tyrosine phosphorylation	7	0.163628	1.03E-07
	GO:0042127	regulation of cell proliferation	7	0.163628	3.16E-07
	GO:0030168	platelet activation	5	0.116877	2.78E-05
	GO:0050900	leukocyte migration	4	0.093502	8.61E-04
	GO:0045909	positive regulation of vasodilation	3	0.070126	9.12E-04
5	GO:0032229	negative regulation of synaptic transm	3	12	2.93E-05
	GO:0019433	triglyceride catabolic process	3	12	5.76E-04
	GO:0060079	excitatory postsynaptic potential	3	12	7.23E-04
	GO:0042157	lipoprotein metabolic process	3	12	0.001334
	GO:0001666	response to hypoxia	4	16	0.001825
	GO:0001523	retinoid metabolic process	3	12	0.003403
	GO:0042632	cholesterol homeostasis	3	12	0.003739
	GO:0050728	negative regulation of inflammatory re	3	12	0.00564
	GO:0033004	negative regulation of mast cell activa	2	8	0.005705
	GO:0007399	nervous system development	4	16	0.007666
	GO:0010886	positive regulation of cholesterol stora	2	8	0.009964
	GO:0051001	negative regulation of nitric-oxide syn	2	8	0.011379
	GO:0051967	negative regulation of synaptic transm	2	8	0.012793
	GO:0042953	lipoprotein transport	2	8	0.021234
	GO:1904707	positive regulation of vascular smooth	2	8	0.021234
	GO:0030301	cholesterol transport	2	8	0.022635
	GO:0010744	positive regulation of macrophage der	2	8	0.022635
	GO:0042310	vasoconstriction	2	8	0.024033
	GO:0048169	regulation of long-term neuronal syna	2	8	0.025429
	GO:0001935	endothelial cell proliferation	2	8	0.025429
	GO:0045777	positive regulation of blood pressure	2	8	0.028216
	GO:0042127	regulation of cell proliferation	3	12	0.028424
	GO:0033344	cholesterol efflux	2	8	0.03515
	GO:0070328	triglyceride homeostasis	2	8	0.036531
6	GO:0055114	oxidation-reduction process	5	83.33333	7.43E-06
8	GO:0055114	oxidation-reduction process	5	1	1.53E-06
	GO:0009258	10-formyltetrahydrofolate catabolic pr	2	0.4	4.76E-04
9	GO:0055114	oxidation-reduction process	6	85.71429	3.12E-07

	GO:0043065	positive regulation of apoptotic proce:	5	0.270856	4.41E-05
	GO:0002576	platelet degranulation	4	0.216685	4.74E-05
	GO:0043547	positive regulation of GTPase activity	5	0.270856	5.06E-04
	GO:0000165	MAPK cascade	4	0.216685	7.44E-04
	GO:0070588	calcium ion transmembrane transport	3	0.162514	0.003138
	GO:0043524	negative regulation of neuron apoptot	3	0.162514	0.003844
**10**	GO:0007268	chemical synaptic transmission	3	0.162514	0.012217
	GO:0048169	regulation of long-term neuronal syna	2	0.108342	0.012792
	GO:0007597	blood coagulation, intrinsic pathway	2	0.108342	0.012792
	GO:2000463	positive regulation of excitatory posts	2	0.108342	0.014204
	GO:0035235	ionotropic glutamate receptor signalin	2	0.108342	0.017022
	GO:0060079	excitatory postsynaptic potential	2	0.108342	0.019833
	GO:0001975	response to amphetamine	2	0.108342	0.021937
	GO:0045429	positive regulation of nitric oxide bios	2	0.108342	0.03031
	GO:0000165	MAPK cascade	3	0.303644	0.002351
	GO:0031293	membrane protein intracellular domai	2	0.202429	0.005349
	GO:0043066	negative regulation of apoptotic proce	3	0.303644	0.006939
	GO:0008284	positive regulation of cell proliferatio	3	0.303644	0.007269
**11**	GO:0001755	neural crest cell migration	2	0.202429	0.013329
	GO:0097190	apoptotic signaling pathway	2	0.202429	0.020965
	GO:0033209	tumor necrosis factor-mediated signal	2	0.202429	0.034649
	GO:0001934	positive regulation of protein phosphc	2	0.202429	0.037252
	GO:0055114	oxidation-reduction process	3	1.456311	0.001241
**14**	GO:0016125	sterol metabolic process	2	0.970874	0.0025
	GO:0006695	cholesterol biosynthetic process	2	0.970874	0.004521

Genes	Fold Enrichment	Bonferroni
TGFB1, CSF2, STAT3, PIK3CD, NGF, MAPK14, T	15.08037719	1.43E-10
SRC, STAT3, IGF1, NGF, MT3, MMP9, EGFR, REI	10.31182935	1.97E-10
IL6, HSP90AA1, IL1B, AKT1, PTGS2, ESR1, TNF,	51.68536252	8.72E-09
HSP90AA1, NOS3, IL1B, AKT1, CALM3, CALM1,	75.98190045	1.16E-08
APP, TF, TGFB1, SERPINE1, CALM3, PLG, TIMP	26.37235865	1.44E-08
IL6, JUN, STAT1, AKT1, IGF1, PTGS2, TNF, FGF2	37.04117647	1.46E-07
AR, IL6, CD40, TGFB1, PLA2G1B, IL1B, CAT, TL	20.42370632	1.88E-07
CD40, TGFB1, CXCR4, PIK3CD, PTGS2, TNF, PIK	9.773397486	3.73E-07
IL6, CD40, PIK3CA, SRC, MAPK1, AKT1, F2, PIK	19.3258312	2.84E-05
JUN, FLT1, PIK3CA, NOS3, MMP2, SERPINE1, KI	12.18095489	2.87E-05
AKT1, CALM3, APOE, CALM1, ESR1, CALM2	67.34759358	3.49E-05
PIK3CA, ERBB2, MAPK1, PIK3CD, AKT1, FGF2,	25.32730015	3.97E-05
CASP9, TGFB1, CASP8, CASP3, STAT3, CAT, PT	21.70911441	1.17E-04
TGFB1, CREB1, NOS2, CASP3, MMP2, CAT, CXC	12.92134063	6.46E-04
HSP90AA1, FLT1, PIK3CA, SRC, KDR, MAPK14,	24.00816993	6.57E-04
IL6, CREB1, STAT3, REL, AKT1, APOE	37.04117647	8.26E-04
TGFB1, AKT1, MAPK14, FGF2, VEGFA	64.98452012	0.001379722
JUN, PIK3CA, BDNF, BCL2, APOE, NGF, MT3, B	14.96613191	0.001494006
FLT1, KDR, AKT1, MAPK14, VEGFA	53.68286445	0.003111739
JUN, STAT1, BCL2, REL, TIMP1, BCL2L1	28.49321267	0.003139626
FLT1, NOS3, IL1B, SERPINE1, KDR, FGF2, VEGF	15.03120205	0.010292488
FLT1, IL1B, MT3, VEGFA	61.73529412	0.051377141
CD40, CASP8, TLR9, REL, TNF, RELA, TNFRSF1	10.73657289	0.067740884
IL1B, TLR9, PTGS2, TNF	47.03641457	0.116172164
APP, BCL2, GAPDH, BCL2L1	29.93226381	0.387130487
BCL2, AKT1, MT3, BCL2L1	27.4379085	0.470220002
APP, AKT1, APOE, PTGS2, EGFR	11.22459893	0.791336368
PLA2G1B, IL1B, PIK3CD, PIK3CG	14.96613191	0.976300721
JUN, TGFB1, CREB1, SRC	10.73657289	0.999929409
PIK3CA, STAT1, NOS3	25.54563895	0.999931703
IL6, ERBB2, FGF2	23.89753321	0.999982156
PTAFR, ADRA1D, AVP, HTR2A, ADRA1B, ADRA	108.5689655	1.07E-06
HCK, CHRM1, FLT4, CRH, ADRA1D, AVP, HTR2	11.18306941	3.92E-04
CHRM3, CHRM1, CDK5, NPY, CHRM5, ADRA1D	12.65649143	0.001090929
PRKCG, GRM5, CDK5, NPY, CRH, HTR2A, GRM	16.88850575	0.00143617
CHRM3, CHRM1, CHRM5	248.1576355	0.034135276
ADRA1D, FPR2, AVP, ADRA1B, ADRA1A	21.60576428	0.041532441
CHRM3, HTR2A, ADRA1A	96.50574713	0.221378982
CHRM3, CHRM1, PTAFR, ADRA1D, FPR2, ADRA	5.152698401	0.285183307
GRM5, LRRK2, GRM1	75.52623688	0.337480862
PRKCG, CRH, S100B	48.25287356	0.636524517
CHRM3, CHRM1, CHRM5	46.94874185	0.656587221

ABCA1, CETP, NR1H2, NR1H3	258.3384615	8.82E-05
ABCA1, LRP1, NR1H2, NR1H3	239.8857143	1.12E-04
CSTB, NR1H2, NR1H3, IDE	159.9238095	4.08E-04
ABCA1, NR1H2, NR1H3	419.8	0.004580295
NR1H2, NR1H3, SREBF2	279.8666667	0.010935573
ABCA1, CETP, NR1H2, NR1H3	52.475	0.012317064
ABCA1, CETP, LRP1	66.28421053	0.189939591
ABCA1, CETP, SREBF2	37.04117647	0.488048267
LYN, BCR, IL5, ERBB3, FYN, FGF1, MET	28.45412733	6.72E-05
JUND, ERBB3, PLAU, LCK, AGTR1, FYN, AGT	23.53233233	2.07E-04
LYN, SYK, VWF, LCK, FYN	27.04025765	0.018003253
LYN, MMP1, LCK, FYN	20.39101396	0.430699945
HMOX1, NOS1, AGT	64.33716475	0.449458741
CNR2, ADORA1, HTR1B	335.84	0.014006428
LPL, APOA1, APOB	80.6016	0.242357018
PPP3CA, ADORA1, SNCA	71.96571429	0.294476898
LPL, APOA1, APOB	53.02736842	0.474437682
F7, PRKCB, ADORA1, HSP90B1	15.62046512	0.585320852
LPL, APOA1, APOB	33.03344262	0.806608637
LPL, APOA1, APOB	31.485	0.835627575
CNR2, PRKCD, APOA1	25.50683544	0.934539495
CNR2, CNR1	335.84	0.936569822
CHRM2, GAL, ADORA1, APOB	9.361393728	0.975509457
LPL, APOB	191.9085714	0.991986348
CNR2, CNR1	167.92	0.995979258
ADORA1, HTR1B	149.2622222	0.997982729
PRKCB, APOB	89.55733333	0.999967853
ERN1, HTR1B	89.55733333	0.999967853
APOA1, APOB	83.96	0.999983876
LPL, APOB	83.96	0.999983876
HTR1A, HTR1B	79.02117647	0.999991913
SYP, SNCA	74.63111111	0.999995944
ERN1, APOA1	74.63111111	0.999995944
CNR1, ADORA1	67.168	0.99999898
BLK, FGR, YES1	10.89210811	0.99999908
APOA1, APOB	53.7344	0.999999968
LPL, APOA1	51.66769231	0.999999984
CBR1, ALDH1A3, ALDH1A2, CRYZ, CBR3	23.63738739	4.46E-04
ALDH1L1, AKR1B10, AKR1B15, AKR1A1, ALDH	28.36486486	2.91E-05
ALDH1L1, ALDH1L2	3358.4	0.00901242
RDH8, MAOA, ALDH1A1, AKR1B1, AOX1, HSD1	24.31274131	1.28E-05

TOP2A, GRIN2A, PSEN1, CLU, GRIN1	21.52820513	0.012807144
SERPINA3, APOH, A2M, CLU	50.16280807	0.013736079
GRIN2A, DLG4, A2M, FGFR3, GRIN1	11.43090538	0.1373865
GRIN2A, DLG4, FGFR3, GRIN1	19.72049325	0.195408518
GRIN2A, PSEN1, GRIN1	32.56367162	0.60055057
HSP90AB1, PSEN1, GRIN1	29.35664336	0.675231951
GRIN2A, DLG4, GRIN1	16.14615385	0.972380656
DLG4, GRIN1	143.5213675	0.976700445
APOH, A2M	143.5213675	0.976700445
DLG4, GRIN1	129.1692308	0.984659708
GRIN2A, GRIN1	107.6410256	0.99335124
GRIN2A, GRIN1	92.26373626	0.997118882
GRIN2A, GRIN1	83.33498759	0.998461422
HSP90AB1, CLU	60.07871199	0.999875013
MAPK11, GDNF, ERBB4	32.04580153	0.313815614
NGFR, ADAM17	310.962963	0.57603884
NGFR, GDNF, ERBB4	18.45274725	0.671790067
ADAM17, GDNF, ERBB4	18.01716738	0.688801696
GDNF, ERBB4	124.3851852	0.883167254
NGFR, PRKCA	78.83568075	0.966296011
NGFR, ADAM17	47.43502825	0.996455097
ADAM17, ERBB4	44.07349081	0.997698566
CYP51A1, CYP46A1, LBR	28.36486486	0.013565319
CYP51A1, CYP46A1	533.0793651	0.027155612
CYP51A1, LBR	294.5964912	0.048621711
