# Supplementary material for: Exploring the Oxidative Stress Mechanism of Buyang Huanwu Decoction in Intervention of Vascular Dementia Based on Systems Biology Strategy
Source: Oxid Med Cell Longev. 2021 Mar 3;2021:8879060. doi: 10.1155/2021/8879060 (PMC7953864; doi:10.1155/2021/8879060)
Supplement: Supplementary 6 — Table S4: signaling pathway of MBHD-VD PPI network. [file 8879060.f6.pdf]

Table S4 Signaling Pathway of MBHD-VD PPI Network

Term	Pathway	Count	%	Pvalue
hsa04024	cAMP signaling pathway	42	0.052775	1.3E-13
hsa04066	HIF-1 signaling pathway	28	0.035183	1.16E-12
hsa04210	Apoptosis	22	0.027644	7.63E-12
hsa04722	Neurotrophin signaling pathway	29	0.03644	6.11E-11
hsa04370	VEGF signaling pathway	20	0.025131	4.05E-10
hsa04012	ErbB signaling pathway	21	0.026388	4.69E-08
hsa04010	MAPK signaling pathway	38	0.047749	6.12E-08
hsa04620	Toll-like receptor signaling pathway	23	0.028901	7.15E-08
hsa04151	PI3K-Akt signaling pathway	43	0.054032	1.37E-06
hsa04150	mTOR signaling pathway	13	0.016335	7.32E-05
hsa04064	NF-kappa B signaling pathway	16	0.020105	8.89E-05
hsa04115	p53 signaling pathway	10	0.012565	0.012304
hsa04330	Notch signaling pathway	8	0.010052	0.017089
hsa04310	Wnt signaling pathway	13	0.016335	0.088603

Genes	Fold Enrichment	Bonferroni
PPARA, ATP1B1, ADORA2A, GLI3, ADORA1, GLI1, AKT	3.751110072	3.14838E-11
TF, ERBB2, TIMP1, AKT1, BCL2, HMOX1, SERPINE1, PII	5.15777635	2.82491E-10
PIK3CG, TNF, RELA, CYCS, PIK3CD, BCL2L1, CAPN2, C	6.274898416	1.85404E-09
AKT1, BDNF, BCL2, PIK3CA, CALML5, AKT3, AKT2, PII	4.273586118	1.48593E-08
PRKCA, PIK3CG, PTGS2, PIK3CD, PRKCG, MAPK11, SR(	5.79796873	9.83612E-08
PRKCA, EGFR, PIK3CG, ERBB4, ERBB3, ERBB2, PIK3CD	4.268504565	1.14006E-05
FGFR2, FGFR1, FGFR4, FGFR3, TNF, TGFB1, AKT1, TNF	2.656065517	1.48634E-05
PIK3CG, IL6, TNF, TBK1, RELA, PIK3CD, MAPK11, MAP	3.837051947	1.73768E-05
HSP90AB1, FGFR2, FGFR1, FGFR4, FGFR3, MCL1, BCL2	2.204068403	0.00033359
PRKCA, PIK3CG, AKT1, MAPK1, TNF, PIK3CD, MAPK3,	3.963611382	0.017634492
ICAM1, TNF, LYN, PTGS2, RELA, TNFSF14, CD40, BCL2	3.252193954	0.021376492
CDK1, CASP3, CASP9, CYCS, CASP8, SERPINE1, IGF1, A	2.639373825	0.950635937
NCSTN, NOTCH3, APH1A, PSEN1, PSEN2, APH1B, ADAI	2.947300771	0.984832357
PRKCA, PPARD, DKK1, PSEN1, JUN, GSK3B, MAPK9, PI	1.665865653	1
