# Supplementary material for: Exploring the Oxidative Stress Mechanism of Buyang Huanwu Decoction in Intervention of Vascular Dementia Based on Systems Biology Strategy
Source: Oxid Med Cell Longev. 2021 Mar 3;2021:8879060. doi: 10.1155/2021/8879060 (PMC7953864; doi:10.1155/2021/8879060)
Supplement: Supplementary 7 — Table S5: enrichment analysis of differentially expressed protein PPI network. [file 8879060.f7.pdf]

Table S5 Enrichment analysis of Differentially Expressed protein PPI Network

Category	Term	Pathway	Count	%	Pvalue
iological process	GO:0002526	acute inflammatory response	4	0.087051	1.05E-05
	GO:0006915	apoptotic process	7	0.152339	5.46E-05
	GO:0035234	ectopic germ cell programmed cell de	3	0.065288	9.55E-05
	GO:0032496	response to lipopolysaccharide	6	0.130577	1.65E-04
	GO:0043065	positive regulation of apoptotic proce	6	0.130577	3.93E-04
	GO:0071407	cellular response to organic cyclic co	4	0.087051	0.001539
	GO:0097192	extrinsic apoptotic signaling pathway	3	0.065288	0.001966
	GO:0045944	positive regulation of transcription fro	8	0.174102	0.002542
	GO:0071310	cellular response to organic substance	3	0.065288	0.00269
	GO:0045429	positive regulation of nitric oxide bios	3	0.065288	0.003232
	GO:0010332	response to gamma radiation	3	0.065288	0.003375
	GO:0032755	positive regulation of interleukin-6 pr	3	0.065288	0.003976
	GO:0045893	positive regulation of transcription, D	6	0.130577	0.004013
	GO:0008285	negative regulation of cell proliferatio	5	0.108814	0.005112
	GO:0009408	response to heat	3	0.065288	0.009433
	GO:0070301	cellular response to hydrogen peroxid	3	0.065288	0.009903
	GO:0001660	fever generation	2	0.043526	0.01124
	GO:0001666	response to hypoxia	4	0.087051	0.01401
	GO:0032308	positive regulation of prostaglandin se	2	0.043526	0.016814
gnaling pathwa	rno05332	Graft-versus-host disease	5	0.108814	1.69E-05
	rno05020	Prion diseases	4	0.087051	6.51E-05
	rno05321	Inflammatory bowel disease (IBD)	4	0.087051	4.96E-04
	rno05140	Leishmaniasis	4	0.087051	6.70E-04
	rno05164	Influenza A	5	0.108814	6.85E-04
	rno04940	Type I diabetes mellitus	4	0.087051	7.85E-04
	rno05152	Tuberculosis	5	0.108814	8.66E-04
	rno04640	Hematopoietic cell lineage	4	0.087051	9.79E-04
	rno05323	Rheumatoid arthritis	4	0.087051	0.001283
	rno04932	Non-alcoholic fatty liver disease (NA	4	0.087051	0.006795
	rno04623	Cytosolic DNA-sensing pathway	3	0.065288	0.008107
	rno05134	Legionellosis	3	0.065288	0.00839
	rno04621	NOD-like receptor signaling pathway	3	0.065288	0.00839
	rno05133	Pertussis	3	0.065288	0.013496
	rno05168	Herpes simplex infection	4	0.087051	0.014884
	rno05132	Salmonella infection	3	0.065288	0.017224
	rno04620	Toll-like receptor signaling pathway	3	0.065288	0.023092
	rno04010	MAPK signaling pathway	4	0.087051	0.023582

Genes	Fold Enrichment	Bonferroni
IL6, IL1B, DEFB1, IL1A	89.69309463	0.006873571
DLC1, PLEKHF1, PRF1, BAX, SIX1, NR4A1, DDIT4	9.863789778	0.035271767
BAX, IL1B, IL1A	193.4007353	0.060902632
IL6, DIO2, IL1B, NFKBIA, IL1A, TRIB1	11.05147059	0.10278324
IL6, DUSP1, BAX, IL1B, NR4A1, SIRT1	9.155064393	0.228110794
IL1B, NFKBIA, KLF2, SIRT1	16.90935391	0.637129049
BAX, IL1B, IL1A	44.20588235	0.726022724
IL6, SIX1, IL1B, NFKBIA, NR4A1, KLF2, SIRT1, IL1A	4.029181985	0.812662613
BAX, IL1B, NR4A1	37.73672884	0.830077759
IL6, IL1B, KLF2	34.38235294	0.881212248
BAX, IL1B, IL1A	33.63491049	0.89191099
IL6, IL1B, IL1A	30.94411765	0.927318904
IL6, SIX1, IL1B, NFKBIA, NR4A1, KLF2	5.419285052	0.929076586
DLC1, IL6, BAX, IL1B, IL1A	6.894856873	0.965690565
IL6, IL1B, IL1A	19.83597285	0.998043325
IL6, KLF2, SIRT1	19.34007353	0.998568133
IL1B, IL1A	171.9117647	0.99941154
APOLD1, IL1B, IL1A, DDIT4	7.640522876	0.999907076
IL1B, IL1A	114.6078431	0.999985739
PRF1, IL6, IL1B, IL1A, RT1-DA	29.35227273	0.00146931
IL6, BAX, IL1B, IL1A	46.96363636	0.005647876
IL6, IL1B, IL1A, RT1-DA	23.84307692	0.042240931
IL1B, NFKBIA, IL1A, RT1-DA	21.525	0.056630993
IL6, IL1B, NFKBIA, IL1A, RT1-DA	11.32894737	0.057853199
PRF1, IL1B, IL1A, RT1-DA	20.39210526	0.066016973
IL6, BAX, IL1B, IL1A, RT1-DA	10.64423077	0.072570867
IL6, IL1B, IL1A, RT1-DA	18.9	0.081716016
IL6, IL1B, IL1A, RT1-DA	17.22	0.105696944
IL6, BAX, IL1B, IL1A	9.566666667	0.447434073
IL6, IL1B, NFKBIA	20.75625	0.507460522
IL6, IL1B, NFKBIA	20.39210526	0.519519413
IL6, IL1B, NFKBIA	20.39210526	0.519519413
IL6, IL1B, IL1A	15.92260274	0.693388448
IL6, IL1B, NFKBIA, RT1-DA	7.175	0.728730719
IL6, IL1B, IL1A	14.00421687	0.779432849
IL6, IL1B, NFKBIA	11.98298969	0.868998469
DUSP1, IL1B, NR4A1, IL1A	6.030350195	0.874592625
